# Optimization and model reduction in the high dimensional parameter space of a budding yeast cell cycle model

**DOI:** 10.1186/1752-0509-7-53

**Published:** 2013-06-28

**Authors:** Cihan Oguz, Teeraphan Laomettachit, Katherine C Chen, Layne T Watson, William T Baumann, John J Tyson

**Affiliations:** 1 Department of Biological Sciences, Virginia Tech, Blacksburg, Virginia 24061, USA; 2 Bioinformatics and Systems Biology Program, School of Bioresources and Technology, King Mongkut’s University of Technology Thonburi, Bangkok 10150, Thailand; 3 Departments of Computer Science and Mathematics, Virginia Tech, Blacksburg, Virginia 24061, USA; 4 Department of Electrical and Computer Engineering, Virginia Tech, Blacksburg, Virginia 24061, USA

**Keywords:** Optimization, Budding yeast, Cell cycle, ODE model, Model reduction, Phenotypic constraints, Latin hypercube sampling, Differential evolution, Sensitivity analysis, Phenotype competition

## Abstract

**Background:**

Parameter estimation from experimental data is critical for mathematical modeling of protein regulatory networks. For realistic networks with dozens of species and reactions, parameter estimation is an especially challenging task. In this study, we present an approach for parameter estimation that is effective in fitting a model of the budding yeast cell cycle (comprising 26 nonlinear ordinary differential equations containing 126 rate constants) to the experimentally observed phenotypes (viable or inviable) of 119 genetic strains carrying mutations of cell cycle genes.

**Results:**

Starting from an initial guess of the parameter values, which correctly captures the phenotypes of only 72 genetic strains, our parameter estimation algorithm quickly improves the success rate of the model to 105–111 of the 119 strains. This success rate is comparable to the best values achieved by a skilled modeler manually choosing parameters over many weeks. The algorithm combines two search and optimization strategies. First, we use Latin hypercube sampling to explore a region surrounding the initial guess. From these samples, we choose ∼20 different sets of parameter values that correctly capture wild type viability. These sets form the starting generation of differential evolution that selects new parameter values that perform better in terms of their success rate in capturing phenotypes. In addition to producing highly successful combinations of parameter values, we analyze the results to determine the parameters that are most critical for matching experimental outcomes and the most competitive strains whose correct outcome with a given parameter vector forces numerous other strains to have incorrect outcomes. These “most critical parameters” and “most competitive strains” provide biological insights into the model. Conversely, the “least critical parameters” and “least competitive strains” suggest ways to reduce the computational complexity of the optimization.

**Conclusions:**

Our approach proves to be a useful tool to help systems biologists fit complex dynamical models to large experimental datasets. In the process of fitting the model to the data, the tool identifies suggestive correlations among aspects of the model and the data.

## Background

The challenges facing molecular systems biologists include the development of accurate mathematical models of complex biological processes [1], the elucidation of design principles that control biological behavior [2], and the generation of new insights into biology that are not apparent solely from experimental studies [3]. A common mathematical method to address these challenges is dynamical systems theory [4,5], the use of nonlinear ordinary differential equations (ODEs) to describe the way networks of biochemical reactions change in time. By comparing the temporal development of the model under conditions that simulate a variety of experimental protocols with the observed behavior of the biological system under the same conditions, one can evaluate how well or poorly the mathematical model performs.

Our focus in this study is parameter estimation of a nonlinear and high-dimensional ODE model (>100 model parameters) that is constrained by a large number of dissimilar experimental observations. The non-differentiable nature of our objective function (described in the next section) led to our choice of a stochastic global optimization approach [6,7] that relies on an evolutionary search, namely differential evolution (DE) [8], starting from a diverse population of parameter vectors scattered over a feasible region of parameter space. DE is a popular global optimization method due to its efficiency and simplicity. However, we should mention that a recent novel (yet more complex) algorithm outperformed DE in multiple optimization tasks with large scale systems biology models due to extensive local search capability [9] that is lacking in the simplest form of DE. For a recent comprehensive review regarding the application of DE and other metaheuristic optimization techniques in systems biology, we refer the reader to [7].

Parameter estimation is not only about finding an “optimal” set of parameter values for fitting a collection of experimental observations. During the course of the global optimization procedure, we expect to find many different parameter vectors that do equally well (or nearly as well) as the best one. Working with this sample of “quite good” sets of parameter values, we can quantify how well the experimental data constrain individual parameter values. We can distinguish critical parameters (highly constrained by the data) from irrelevant parameters (those that have little bearing on optimization of the objective function) [10]. We can distinguish those experimental results that provide the most information about the underlying model from those that provide the least, and we can design new experiments that will provide the most new information about the underlying molecular regulatory system [11-13]. All these types of information can be very useful in refining and extending the model [14].

Our research group has been interested for many years in the molecular mechanisms controlling the cell division cycle of budding yeast. The main events of the cell cycle (DNA synthesis and mitosis) are controlled in budding yeast, and indeed in all eukaryotic cells, by a family of protein kinases called cyclin-dependent kinases (CDKs) [15]. We have built comprehensive and accurate models of the periodic activation of CDKs, based on nonlinear ODEs describing the underlying biochemical reaction network [16]. The models are used to understand how CDKs control cell cycle progression in normal (“wild type”) yeast cells, and also how cell cycle progression is altered in yeast strains harboring mutations in genes of the CDK control system. Each mutant strain is characterized as “viable” or “inviable”. A viable mutant cell is able to grow and divide despite its altered control system, whereas an inviable mutant cell is blocked at some stage of the cell cycle and eventually dies. The wild type strain is, of course, viable.

**Figure 1:**
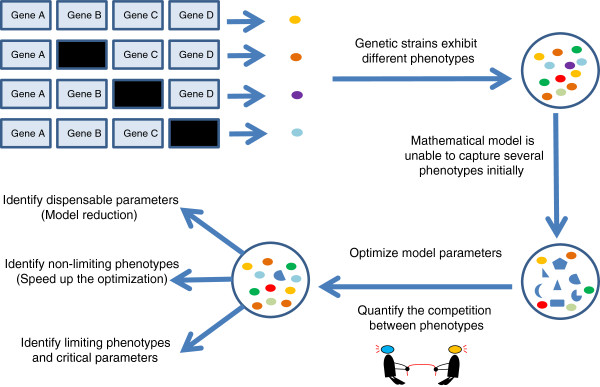
Schematic of the parameter optimization and model reduction approach.

In this study, we present an optimization procedure to maximize the number of strains for which the model correctly captures viability or inviability (Figure 1). Our approach applies quite generally to exploring the parameter space of a high-dimensional ODE model in order to maximize how well the model accounts for a large collection of experimental data, particularly in cases where the objective function may not depend smoothly on parameter values. We start with an initial region of parameter space (we will refer to this region as a “hypercube”—although we do not mean to imply that the edge lengths of the box are identical) where we think a reasonable solution must lie. We use Latin Hypercube (LH) sampling to provide a starting sample of parameter vectors, widely distributed over the initial hypercube [17], that are consistent with “wild type” (WT) viability. Next, we use DE that starts from this population of WT parameter vectors and searches for new combinations of parameter values that satisfy an ever larger number of experimental constraints (mutant phenotypes). Our approach not only produces a sample of parameter assignments that provide good fits to the collection of mutant phenotypes but it also provides insights about how the model can be reduced without compromising its ability to explain the experimental data. Model reduction, parameter identifiability, and parameter sensitivity are critical, related concepts for the analysis and construction of models. Relevant recent work regarding identifiability and model reduction in systems biology includes implementations of singular value decomposition to reduce models where parameter estimation is posed as a linear regression problem [18,19], computation of confidence intervals by extensive parameter sampling to detect non–identifiable parameters [20], and ranking of parameters by means of sensitivities [21]. Recent reviews on identifiability of systems biology models can be found in [22,23]. Our main contribution is a new methodology for model reduction in the absence of a continuous objective function, unlike the aforementioned works that use a continuous objective based on time-series data. We also propose a novel way to quantify the competition between individual experimental constraints and reduce their number to speed up the optimization process in the context of a discontinuous objective function.

## Methods

### Problem formulation

In this paper, we focus on biochemical reaction networks modeled by nonlinear ODEs. Typical models of these networks that are considered high dimensional, at the present time, consist of 10–100 ODEs defined in terms of ∼100 (or more) rate constants and other numerical parameters. The models are developed in light of and the parameters are constrained on the basis of large collections of experimental data, which characterize the behavior of cells under a wide variety of experimental conditions. The data are rarely replicate measurements of time courses of biochemical variables; the sort of ideal data assumed in many optimization methods. Rather, the data are often a disparate collection of quantitative measurements and qualitative observations on a number of different mutant strains under a wide variety of conditions. In this context, a data-fitting algorithm must be able to search a high-dimensional parameter space for parameter vectors that are consistent with as much of the data as possible. In our case, we characterize the data as a set of *n* constraints. For a specific parameter vector, the model either satisfies the *i*^*th*^ constraint (*o*_*i*_=1) or not (*o*_*i*_=0), and the total objective function that we seek to maximize is $O=\sum_{i=1}^{\text{n}}o_{i}$. The discontinuous, stepwise nature of this objective function prohibits the use of any gradient-based optimization method, even if multiple starting points are used. Therefore, in looking for optimal behavior of the model, we search a region of parameter space stochastically and keep track of all parameter vectors encountered during this search.

Using this collection of optimal (or near optimal) parameter vectors, our second aim is to characterize the roles of specific parameters and specific experiments in the data-fitting exercise. Looking at the sensitivity of experimental constraints with respect to parameter variations, we distinguish “critical” parameters, which have strong effects on the total objective function, from “dispensable” parameters, which have little or no effect on the total objective function. We also distinguish “fragile” phenotypes, which are most often broken (i.e., incorrectly simulated) under parameter variations, from “robust” phenotypes, which are correctly simulated even when parameter values are widely perturbed. These distinctions provide insights into the relationships between the model and the data, and they also allow us to reduce the complexity of the model (by eliminating dispensable parts) and the computational demands of the algorithm. Finally, we look at competition (negative correlations) between experimental constraints (phenotypes). If two phenotypes compete with each other, then it is difficult for the model to account simultaneously for both. The list of most competitive phenotypes suggests places where the structure of the model may be incorrect or the experimental observations may be suspect.

### A mathematical model of the budding yeast cell cycle

The cell cycle is the sequence of events by which a growing cell replicates all its components and divides them more-or-less equally between two daughter cells, so that the daughters inherit all the machinery and information necessary to repeat the process [15,24]. The most important components that need to be accurately replicated in the mother cell and precisely partitioned to the progeny cells are the DNA molecules of the cell’s genome. New DNA molecules are synthesized during S phase and distributed to progeny nuclei during M phase (mitosis). S and M phases are separated by two gaps: G1 (DNA unreplicated) and G2 (DNA replicated). The ordered sequence of cell cycle phases, G1-S-G2-M, is governed by the periodic activation of CDKs. Activity of CDKs depend on cyclins, which are regulatory proteins that are needed to form active cyclin-CDK complexes. In budding yeast, the earliest CDKs to be activated are Cln1- and Cln2-dependent kinases, which promote the appearance of later cyclins as well as initiating bud emergence. Clb5- and Clb6-dependent kinases are essential for timely DNA synthesis, and somewhat later, Clb1- and Clb2-dependent kinases arise to drive the cell into mitosis. To exit from mitosis and return to G1, all the Clb-cyclins must be cleared from the cell, which is the job of the APC (anaphase promoting complex) in conjunction with its partners, Cdc20 and Cdh1. Some other important components of the control system are: Sic1 (a stoichiometric inhibitor of Clb-dependent kinases), Cdc14 (a phosphatase that opposes the action of CDKs), Net1 (a stoichiometric inhibitor of Cdc14), SBF (a transcription factor for Cln1,2 and Clb5,6), Mcm1 (a transcription factor for Clb2, Swi5 and Cdc20), and Swi5 (a transcription factor for Sic1). All these molecules (and some others we have not mentioned) are involved in a complex biochemical reaction network that controls the periodic activation and inactivation of the CDKs (which drive the cell from G1 phase into and through S-G2-M) and Cdc14-Cdc20-Cdh1-Sic1 (which drive the cell out of mitosis and back to G1).

A mathematical model of this reaction network was developed by Chen et al. [16]. This model consists of 36 ordinary differential equations (with 135 kinetic parameters) and reproduces the biological properties of ∼125 mutant strains of budding yeast. The “properties” include not only viability-inviability of the strains but also average size of cells at division, relative timing of bud emergence, DNA synthesis and mitosis, and the precise phase of arrest of inviable mutants. The Chen-2004 model evolved over the course of about 10 years, as the experimental basis of the model was being discovered by molecular geneticists and as the molecular interactions were translated into differential equations by the mathematical modelers. The parameter values “evolved” along with the model, so that at no time were the modelers faced with the daunting task of fitting a 135-parameter model to a 125-component objective function. In this study, we focus on a new formulation of the Chen-2004 model. This model (a detailed description can be found in [25]) uses a simpler mathematical framework, requiring fewer ODEs (26) and kinetic parameters (126), while improving on the model’s representation of the G1/S transition and exit from mitosis. The starting set of parameter values for the optimization, produced by manual tuning, captured the basic cell cycle characteristics of wild type cells as well as the phenotypes (viable or inviable) of ∼60% (72 out of 119) of the genetic strains. Our goal was to develop an automatic method for finding parameter values that capture nearly all the mutant phenotypes when starting from an educated “initial guess” of parameter values.

We provide descriptions of the 126 model parameters and 26 model variables (Additional file 1: Tables S1 and S2) along with the numerical values of these parameters from the “initial guess” (Additional file 1: Tables S3 and S4). We also provide a C++ subroutine that implements the model along with a Matlab script (Additional file 2), taking as input values the 126 parameters and 26 initial conditions for the model, and giving as output the phenotypes (viable or inviable) of 119 budding yeast strains: WT growing in glucose + WT growing in galactose + 117 cell-cycle mutants growing in glucose or galactose. All 119 strains are listed in Additional file 1: Table S5 along with the parameter changes for each strain with respect to WT conditions. The C code solves the ODEs using Euler’s method with a fixed step size of 0.05 minutes. While there are certainly more sophisticated ODE solvers, this solver was chosen because it easily handles both deterministic and stochastic cases, and also allows direct comparison with previous work on this model [25]. The code first simulates WT cells growing in glucose, using the 26 initial conditions provided on input (“input-ICs”), for a total of 2000 min. If at any time during this simulation cell size (mass) exceeds 25 units, the cell is considered inviable. Otherwise, the program asks: is cell size at the last division is within 5% of the cell sizes at the two previous divisions? If “yes”, then the cell is considered as“viable”. (Note: sometimes, after a period-doubling bifurcation, the model generates periodic cell divisions with size at division oscillating between two values that differ by more than 5%. These cases are considered neither viable nor inviable.) If the WT cell (growing in glucose) is viable, then we record the initial values of all variables just after the last division of the WT cell. These values (the “newborn-ICs”, which bear no relation to the input-ICs) are used for the simulations of all other 118 strains. Each strain simulation is classified as viable or inviable by the same rules applied to the WT simulation. To calculate the value of the objective function for the given set of parameter values and input-ICs, we then sum up 119 values of an indicator function that is 1 if the phenotype (viability or inviability) of the simulated strain is the same as the observed phenotype, and is 0 if the phenotypes are different. The objective function is an integer-valued function that varies from 0 to 119. (When the WT cell growing on glucose is inviable, we use default-ICs to simulate the mutant strains.) Finally, we introduce here the nomenclature of budding yeast genes and mutant alleles. Proteins, such as Cln2, Cdc20 and Sic1, are encoded by wild type genes: *CLN2, CDC20* and *SIC1*. Mutant alleles are indicated by lower case, italicized names: *cln2, cdc20, sic1*. The notation *sic1* Δ means the wild type *SIC1* gene has been deleted from the genome, and the notation *GAL-SIC1* means that the WT *SIC1* gene is being expressed continuously at high level from a galactose-inducible promoter. The meaning of other gene notations used later in this paper can be found in Chen et al. [16] or on our budding yeast web page [26].

### The parameter estimation algorithm

We start our search of parameter space from a point supplied by the modeler (initial guess). We assume that the starting point is a reasonable (but not particularly good) estimate of the parameters. That is, the starting parameter values are consistent with some but not all experimental constraints, and we expect that a much better parameter vector is in the neighborhood. In our case, the initial guess is consistent with 60% of the mutant phenotypes, and we plan to search in a hypercube (e.g. ±40% or ±90%) around the starting point. First, we explore this domain by Latin Hypercube (LH) sampling, as described in detail in the Additional file 3. (LH sampling is commonly used to generate multidimensional samples from a multidimensional distribution [17]). To obtain a “population” of prospective parameter vectors for the next phase of the search, we select from the LH samples only parameter vectors that are consistent with viability of WT cells growing on glucose.

For the second phase of the search, we use differential evolution (DE) to improve the performance of the LH-derived population of parameter vectors [8]. The basic idea behind DE is to allow a population of parameter vectors to evolve over many generations of reproduction and selection. During the reproduction step, each “parental” parameter vector generates an “offspring” parameter vector, which differs from the parent by a process of “diversification”. Then, the parent and its offspring compete with each other: the better vector (the one with the higher value of the objective function) goes on to the next generation, the less good vector is set aside.

To be precise, let **x** be a vector of parameter values, with components *x*_*i*_, *i* = 1,2,…,*D*, where *D* is the dimension of the parameter space. (Note that the vector **x** includes both the 126 kinetic constants in the model and the 26 ODE initial conditions described above; hence, *D*=152. This is another conservative choice on our part; later we will show that the 26 input-ICs have little or no bearing on the ultimate success of the model). As described in the previous section, the objective function *O*(**x**) is an integer-valued function that counts the number of phenotypes that are correctly captured by the model given the parameter values in the vector **x**. (Notice that we sometimes refer to a particular parameter vector as a “set of parameter values”).

During DE, parameter vectors are propagated from generation to generation by processes of diversification and selection. Each generation (indexed by *t* = 0,1,…) consists of *N* parameter vectors **x**_*j*_(*t*), *j* = 1,…,*N*. Hence, the real number *x*_*i*,*j*_(*t*) is the value of the *i*^*th*^ parameter in the *j*^*th*^ parent in the *t*^*th*^ generation. Let **u**_*j*_(*t*) be the parameter vector for the single offspring of the *j*^*th*^ parent in the *t*^*th*^ generation. The components of this vector, *u*_*i*,*j*_(*t*) for *i*=1,…,*D* are constructed in two steps (called “mutation” and “crossover”). Then, given the two parameter vectors **x**_*j*_(*t*) and **u**_*j*_(*t*), a decision is made as to which one is propagated to generation *t*+1.

The specific rules are: 

1. Mutation: First, we create a “mutant” vector **v**_*j*_(*t*) by perturbing a parental parameter vector **x**_*j*_(*t*):

$$v_{j}(t)=x_{j}(t)+F\cdot d_{j}(t).$$

By analogy to biological evolution, we might let the components of **d**_*j*_(*t*) be random perturbations of the parental parameter values. However, we use the strategy of DE, letting the perturbation vector be the difference between the parameter vectors of two additional parents, *j*^′^ and *j*^′′^, chosen at random from the *t*-th generation of parents. (All three parents must be different). In this case, the “mutant vector” is defined by

$${\text{v}}_{j}(t)={\text{x}}_{j}(t)+F\cdot ({\text{x}}_{j^{\prime }}(t)-{\text{x}}_{\mathrm{j\prime \prime}}(t)),$$

where 0 <*F* <1. (We are conservative in our choice of *F* = 0.1). With this definition, perturbations can be large at first, when the population of parental parameter vectors is diverse in terms of individual parameter values, but the size of perturbations will decrease in later generations as the population converges on a nearly common set of parameter values.

2. Crossover: Next we allow for crossover between the parental parameter vector **x**_*j*_(*t*) and the mutant parameter vector **v**_*j*_(*t*). Component-wise, the offspring vector **u**_*j*_(*t*) receives a parameter value from the mutant vector with probability *C* (the “crossover” probability) or from the parent vector with probability 1−*C*:

$$u_{i,j}(t)=\left\{\begin{array}{c} v_{i,j}(t)\;\text{if rand(0,1)}\;\leq C\\ x_{i,j}(t)\;\text{otherwise} \end{array}\right)$$

i=1,2,…,Dandj=1,2,…,N,

where rand (0,1) is a random number chosen uniformly from the interval [0,1]. We choose *C*=0.5 so that neither parental values nor mutant values are given an advantage during the crossover step.

3. Selection: The objective function determines whether, **x**_*j*_(*t*) or **u**_*j*_(*t*), passes on to the next generation. There are two possibilities here. The “greedy” algorithm says that the offspring replaces its parent if it is superior:

$$x_{j}(t+1)=\left\{\begin{array}{c} u_{j}(t)\;\text{if}\;\;O(u_{j}(t))>O(x_{j}(t)),\\ x_{j}(t)\;\text{otherwise.} \end{array}\right)$$

With the “non-greedy” version, the selection condition is *O*(**u**_*j*_(*t*)) ≥ *O*(**x**_*j*_(*t*)).

In a few hundred generations, DE produces an elite set of parameter vectors that reproduce the behavior of nearly all the experimental constraints despite the suboptimal performance of the starting point of the optimization.

All computations were performed in the Advanced Research Computing lab at Virginia Tech. The computational time was ∼4 minutes for a single generation of DE (19 parameter vectors and 119 simulations per vector) and ∼20 minutes for 100 LH samples (12 seconds per sample). Computation time could be significantly reduced by parallel computing, e.g., 500 generations of DE, which took ∼33 hours in our code, could be completed in ∼1 hour by using 33 processors in parallel. Such a reduction may be important in the future when we impose additional constraints on the model.

In concluding this section we note that, in addition to varying the values of *C* and *F*, there are other diversification and selection strategies that could be implemented in DE [27]. In this study we are served well by the most basic mutation and crossover strategies, with conservative values of *C* and *F*. Investigation of the effects of varying *C*, *F*, and mutation and crossover strategies is beyond the scope of this paper.

## Results and discussion

### Rapid evolution to high-scoring parameter vectors

**Figure 2:**
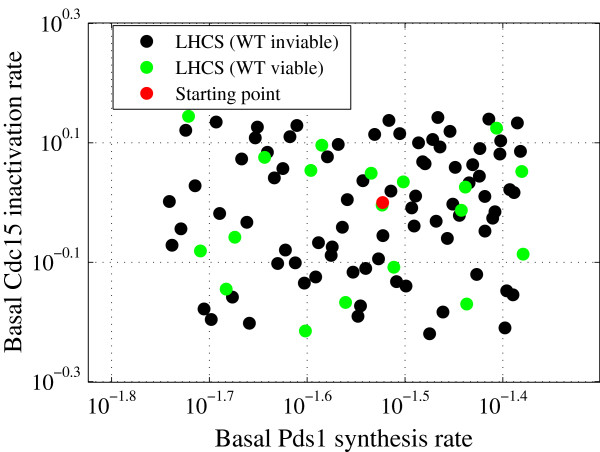
**First step of the optimization: Latin Hypercube sampling.** Feasible and infeasible parameter vectors during LH sampling (green and black dots), and the starting point of the parameter search (red dot). This is a projection of the whole parameter space onto the axes of two model parameters (basal Pds1 synthesis and basal Cdc15 inactivation rates).

**Figure 3:**
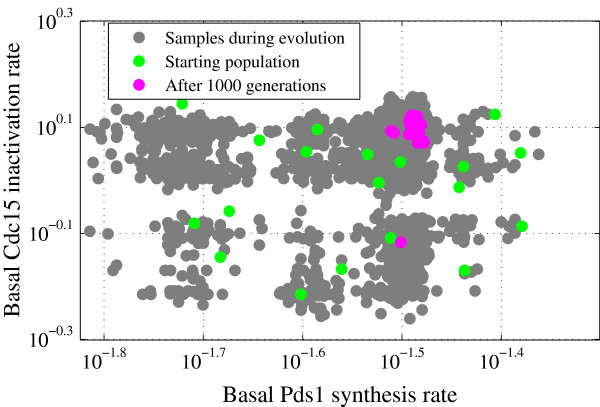
**Second step of the optimization: differential evolution.** Starting population (green dots, from Figure 2), final population (pink) and all samples (grey dots) taken during a DE run with population size *N* = 19 and 1000 generations. The *x* and *y* axes represent the same model parameters as in Figure 2.

**Figure 4:**
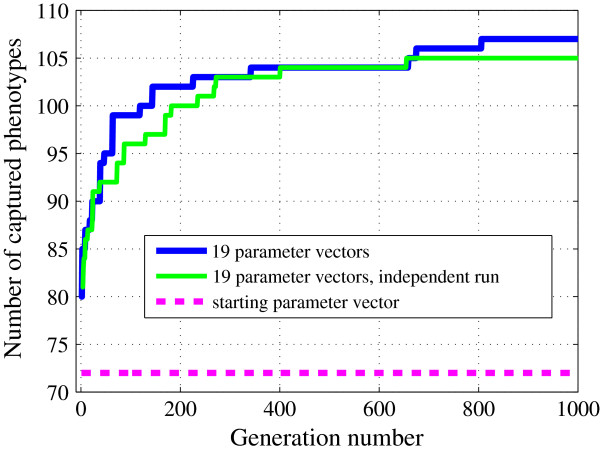
**Evolution of the objective function during optimization.** Increase in the value of the objective function (number of mutants captured) during DE runs with population size *N* = 19. Pink line: Model performance for the initial guess for the parameter vector (*O* = 72 hits; *O*_*max*_ = 119). Green and blue lines: Number of mutants captured in two independent DE runs with the same initial population of 19 parameter vectors. Different random number sequences in mutation and crossover operations in these runs.

We performed LH sampling around the starting point (initial guess) in a hypercube formed by ±40% perturbations on each parameter value. To create 100 sample points inside this hypercube, each parameter range is divided into 100 subintervals (see Additional file 3). Of the 100 samples, 19 reproduced WT viability in glucose (Figure 2). These 19 parameter vectors were used as the starting population, **x**_*j*_(0),*j* = 1,…,19, for the DE algorithm (Figure 3). We use a greedy selection algorithm at first and see a steady increase in the objective function (“number of hits”) during the evolution process, (Figure 4). Here, the starting parameter vector gets 72 hits (out of 119 max, 60% success), and the best score among the original 19 parameter vectors generated by LH sampling was 80 hits. During DE, the objective function increases by ∼50% to 107 hits. An independent DE run (Figure 4) with the same initial population of parameter vectors, but a different sequence of random numbers used in mutation and crossover operations, reached 105 hits. This variability of success comes, presumably, from the stochastic nature of DE and the highly nonlinear structure of our model.

### Varying the settings of the optimization procedure

**Figure 5:**
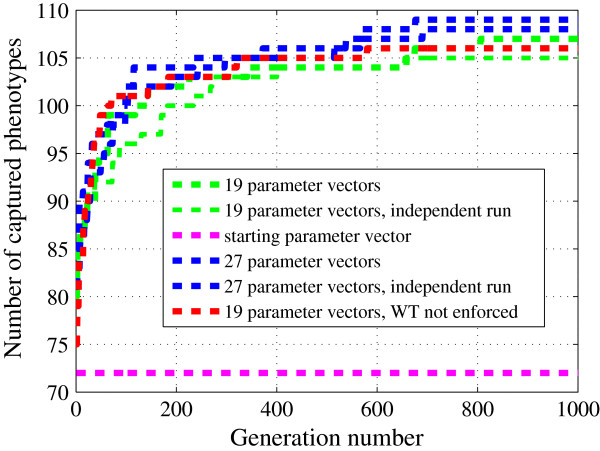
**Effects of population size and criterion used in LH sampling on the optimization.** Increase in the number of mutants captured during independent DE runs for population sizes *N* = 19 (green lines) and *N* = 27 (blue lines). Pink line: Model performance for the initial guess for the parameter vector (*O* = 72 hits; *O*_*max*_ = 119). Red line: WT viability in glucose is not enforced during LH sampling phase.

The initial phase of LH sampling can be quite variable in its outcome. For example, when we resampled the ±40% hypercube around the initial guess with 50 LH samples, we found *N*=27 parameter vectors consistent with wild type viability in glucose. Nonetheless, running DE on this larger starting population did not make a significant difference in the final value of the objective function (109 hits for *N*=27 versus 107 hits for *N*=19), see Figure 5. Furthermore, when we generated 19 LH samples (±40%) without regard to WT viability, the DE algorithm reached a maximum of 105 hits, indicating that the algorithm’s success is not highly dependent on the initial population’s ability to capture wild type viability in glucose. However, we should note that the center of the LH (the initial guess) is known to be a reasonably good starting point (72 hits). Hence, we can expect that there are good solutions to the optimization problem within the LH even if none of the initial 19 parameter vectors are particularly good. In fact, we formed four independent sets of 19 parameter vectors from the 81 vectors (out of 100 LH samples) that failed to reproduce wild type viability. For these four starting populations, DE converged to 100, 104, 106, and 107 hits in 1000 generations.

**Figure 6:**
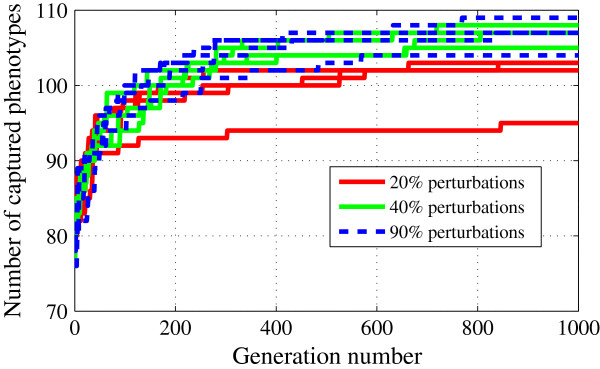
**Effects of hypercube size used in LH sampling on the optimization.** Increase in the number of mutants captured with initial populations generated by ±20% (red lines), ±40% (green lines) and ±90% (blue lines) perturbations around the starting parameter vector. Once again, the independent runs for each perturbation setting have identical initial populations, but different random number sequences used in the mutation and crossover operations in DE.

We also investigated how the size of the LH affects the performance of DE, by starting with hypercubes generated by ±20%, ±40% and ±90% perturbations around the initial guess. In each case, we started the DE with a population of 19 parameter vectors generated by LH sampling without enforcing the viability of wild type cells in glucose. As illustrated in Figure 6, 40% and 90% perturbations gave similar results after three DE runs (105–108 hits and 104–109 hits, respectively). On the other hand, 20% perturbations gave lower performance (95–103 hits after 1000 generations). In fact, 50 independent DE runs (for 500 generations) with 20% perturbations showed a similar trend (illustrated in Additional file 1: Table S6), whereas 40% perturbations performed best (convergence curves of these runs are shown in Additional file 4: Figure S1). These results indicate that if there is not enough variability in the LH samples used to initiate DE (e.g., the hypercube is not big enough), then DE performs suboptimally.

**Figure 7:**
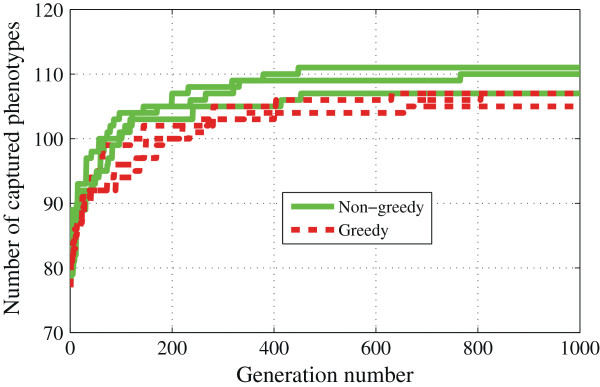
**Effects of selection strategy on the optimization.** Performance comparison of greedy and non-greedy selection rules. Solid lines: non-greedy selection. Dashed lines: greedy selection. All six runs start with the same initial population but with different random number sequences in mutation and crossover operations.

Another variation in the algorithm is the criterion we use for deciding when a trial parameter vector can replace a parent. As shown in Figure 7, compared to the greedy selection criterion we have been using, non-greedy selection gives faster convergence to good solutions, although the final success of the two strategies is about the same. Defining convergence as the first generation in a run when the algorithm reaches 100 hits, we find that greedy runs converge in ∼150 generations (median value) whereas non-greedy runs reach 100 hits in ∼70 generations (median value).

We also investigated the effect of the starting point on the performance of our optimization procedure. Starting from the initial guess, we ran DE for 200 generations without enforcing any improvement (random walks) and randomly picked a parameter vector from the last generation as a potential starting point. Repeating this process two more times gave us three new starting points for optimization with 54, 69 and 57 hits. These new starting points differed from the initial guess by ∼25% across all parameter values. Starting from these three points, we used both 40% and 90% LH sampling, followed by DE with non-greedy selection. The success rates of these runs (Table 1) indicate that the performance of our optimization procedure is not highly dependent on the quality of the starting point.

**Table 1:** Variations in the starting point of optimization

**Initial search point**	**Initial # hits**	**Final # hits**	**Final # hits**
**indicator**		**(40% LH)**	**(90% LH)**
0	72	105–108	104–109
		(3 runs, Figure 6)	(3 runs, Figure 6)
1	69	107	103
2	57	104	107
3	54	101	106

Optimization results with different initial search points used in LH sampling.

A further variation of our parameter estimation study involved stopping DE at a particular generation, grabbing the best population member, resampling around it with the LH approach and continuing DE. Here, we focused on the ten worst performing runs in Additional file 4: Figure S1. The average number of hits among these runs was 101.80, four less than the average of the 50 runs. When we performed LH sampling around the best performing population member within each run (with 40% perturbations) at generation 500 and continued DE for an additional 500 generations, the average number of hits increased to 107.80. In comparison, continuing DE for the same number of generations without any resampling led to an average of 103.9. Using the LH resampling approach after 100 generations (instead of 500) and continuing DE for 500 generations carried the average number of hits from 96.30 to 105.90, which is approximately equal to the average from the 50 runs in Additional file 4: Figure S1. On the other hand, continuing DE for 500 generations without resampling resulted in an average of 102.3 hits. From these results, we conclude that this resampling strategy is useful to improve suboptimal runs.

### Robustness of the model

As an indicator of the model’s robustness with respect to the phenotype of a particular yeast strain we introduce the “acceptance ratio” for an experimental constraint, which is simply the fraction of sample parameter vectors that are consistent with an observed phenotype [28,29]. For example, consider the 100 LH samples in Figure 2. The acceptance ratio of “WT viability in glucose” is 19%. That is, the model is not particularly robust at accounting for the viability of WT yeast cells if parameter values are chosen more-or-less randomly in a hypercube of parameter space, even if this box is known to contain quite good parameter vectors. Since the acceptance ratio for WT viability among a small number of LH samples is known to be highly variable, we generated a set of 19,000 LH samples in the ±40% hypercube. Within this collection, “WT viability in glucose” has a 25% acceptance ratio.

By comparison, if we optimize overall success rate, then we find that WT viability is an extremely robust property of the model. For example, we maximize the total number of hits on a population of 19 parameter vectors over 1000 generations, without enforcing WT viability in the LH and DE stages. In the collection of 19,000 samples (trial parameter vectors, some of which did not replace the parents), the acceptance ratio for “WT viability in glucose” was 0.9964.

**Figure 8:**
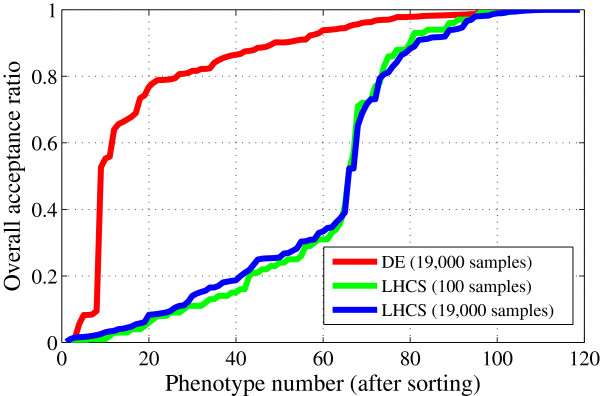
**Overall acceptance ratios of phenotypes with DE and LH sampling.** The *x* axis represents the phenotype number after sorting phenotypes with respect to their acceptance ratios. The DE curve (red line) comes from the most successful run (111 hits), where 19 parameter vectors were gradually refined over the course of 1000 generations; for the 19,000 trial parameter vectors generated during this run, the overall acceptance ratio for each phenotype was computed. LH curves come from independently generated 100 and 19,000 LH samples (green and blue lines).

For these three sample sets (19,000 DE samples, 19,000 LH samples and 100 LH samples) we computed the acceptance ratios of all 119 experimental strains and sorted them in ascending order, as shown in Figure 8. Comparing LH performance to DE, we see the power of DE to find regions of high overall model performance in parameter space. The set of 19,000 trial parameter vectors generated during DE is quite robust in accounting for most phenotypes (∼110 phenotypes have an acceptance ratio greater than 0.5). By contrast, for 19,000 LH samples without any selection, only ∼50 strains have an acceptance ratio greater than 0.5. Despite the inability of LH sampling to find very successful parameter vectors, the LH sampling step is essential to provide DE with an initially diverse population that is able to evolve to high scoring parameter vectors. It is also apparent from Figure 8 that most of the difficulties encountered by DE are the consequence of eight strains with very low acceptance ratio (Additional file 1: Table S7). Apparently it is very difficult to parametrize the model to fit any of these eight strains without disturbing the fit to many other strains, suggesting that there may be some missing interactions in the model, or there may be some mistaken phenotypes reported in the literature, or both.

### Competition between the experimental constraints

Given the high dimensionality of the model, one may think that it is relatively easy to capture the biological behavior of the majority of the mutants [30]. However, that is not the case when mutant phenotypes compete against each other to be correctly simulated by the model, as suggested by the existence of eight “low acceptance” phenotypes in Figure 8 (red line). To quantify this competition, we keep track of the performance of the parameter vectors generated during the LH and DE phases of the optimization procedure. With *m* as the total number of parameter vectors and *n* as the total number of phenotypes, we define the *m*×*n* acceptance matrix:

$$A=\left[\begin{array}{llll} A_{1,1} & A_{1,2} & \cdots & A_{1,n}\\ A_{2,1} & A_{2,2} & \cdots & A_{2,n}\\ \vdots & \vdots & \ddots & \vdots \\ A_{m,1} & A_{m,2} & \cdots & A_{m,n} \end{array}\right],$$

where *A*_*i*,*j*_=1 if parameter vector *i* captures phenotype *j*, otherwise *A*_*i*,*j*_=0. Then, we compute the *n*×*n* matrix of correlation coefficients:

$$R_{k,l}=\frac{C_{k,l}}{\sqrt{C_{k,k}C_{l,l}}},$$

where *C*_*k*,*l*_ is the covariance of the acceptance values of phenotypes *k* and *l*:

$$C_{k,l}=\frac{1}{m-1}\overset{\text{m}}{\underset{i=1}{\sum }}(A_{i,k}-{\bar{A}}_{k})(A_{i,l}-{\bar{A}}_{l}).$$

Here, ${\bar{A}}_{l}$ is the acceptance ratio of phenotype *l* among *m* parameter vectors. *R*_*k*,*l*_ quantifies the correlation between the *k*^*th*^ and *l*^*th*^ phenotypes. For each phenotype, we compute an overall correlation value ${\hat{R}}_{k}=\sum_{l=1,k\neq l}^{\text{n}}R_{k,l}$ (*k*=1,2,…*n*), that quantifies the strength of the competition faced by the *k*^*th*^ phenotype during the optimization against the remaining phenotypes.

Next, we identify the strongly anticorrelated mutants and explain how they influence the search for model parameters while we maximize the fraction of the phenotypes that are captured. Our focus is on the DE run that resulted in 111 phenotypes being captured (the blue line in Figure 7, yielding 19,000 parameter vector samples, 22% of which replaced their parents). In addition, we analyze the LH run with 100 samples. Additional file 1: Tables S8 and S9 show the most competitive phenotype pairs in the LH and DE samples. We see that, in all cases, an experimentally viable phenotype is paired with an inviable one. Among LH samples, the majority of competitive phenotype pairs have common mutations. However, this is not the case among DE samples, which suggests that non–intuitive competitions arise when we maximize the number of captured phenotypes in the DE phase.

Among the 3415 top-performing parameter vectors in the DE run with 111 hits, there is a common set of eight phenotypes that are not captured. Seven of these missed phenotypes (#46, 48, 55, 66, 67, 74, and 117 listed in Additional file 1: Table S7) have reasonably high acceptance ratios (0.36–0.94) among the 100 LH samples, but each one is strongly anticorrelated with the remaining phenotypes. To demonstrate this fact, we sorted the 119 $\hat{R}$ values in ascending order from most competitive ($\hat{R}=-8.32$) to least competitive ($\hat{R}=+13.71$). The seven phenotypes under consideration are in the top 22 most competitive phenotypes in the 100 LH samples, as listed in Additional file 1: Table S7. Furthermore, based on their $\hat{R}$ values, these seven phenotypes are in the top eight most competitive phenotypes in the 19,000 DE sample set. Despite their reasonably high overall acceptance ratios in the LH samples, they experienced a large drop in acceptance ratio during DE due to their strong competition with other phenotypes (illustrated in Additional file 1: Table S7).

The eighth missed phenotype (# 12: *cln1* Δ *cln2* Δ *bck2* Δ) has very low acceptance ratio in both 19,000 DE samples (6%) and 100 LH samples (3%). Despite the ability of DE to increase the acceptance ratios of most phenotypes (Figure 8), this phenotype’s acceptance stays low as a result of its competition with the remaining 118 phenotypes during DE. The $\hat{R}$ value of this phenotype is the 6^*th*^ most competitive in the 19,000 DE samples. On the other hand, within the 100 LH samples, strain 12 is non-competitive, as illustrated in Additional file 1: Table S7 (its $\hat{R}$ was ranked 48^*th*^). Additional file 4: Figure S2 shows a comparison of pairwise correlations (*R* values) of phenotype 12 in 100 LH samples versus 19,000 DE samples. There are 35 *R* values above 0.12 in the LH phase, whereas this number drops to zero in the DE phase as majority of phenotypes become competitors of phenotype 12. $\hat{R}$ of phenotype 12 drops from −0.87 to −11.35 as a consequence of this transition. From these observations, we conclude that phenotype 12 is not captured due to its extremely low acceptance ratio among LH samples and also during optimization.

Some of the eight non-captured phenotypes considered here are also among the “most troublesome” strains identified by us during trial-and-error parameter estimation. For example, it is difficult to explain the inviability of strains 66 (*CLB1 clb2* Δ *cdh1* Δ) and 67 (*CLB1 clb2* Δ *pds1* Δ) because the single mutants (*cdh1* Δ and *pds1* Δ) are viable and it is unclear why deletion of *CLB2* from either single mutant should make the strains inviable. We also observed that capturing the viability of very large cells of strain 12 (*cln1* Δ *clb2* Δ *bck2* Δ) jeopardizes the simulations of many other mutants, as confirmed in Additional file 4: Figure S2-b.

Some of the other non-captured phenotypes point out limitations of our model and/or objective function. Strain 74 (*GAL-CDC20*) is inviable because excess Cdc20 overwhelms the mitotic checkpoint mechanism and drives cells into premature anaphase, but our model does not capture this effect, probably because we do not overexpress Cdc20 to a sufficiently high level. Strains 46 (*CLB5-db* Δ *sic1* Δ) and 48 (*GAL-CLB5 sic1* Δ) are viable by our criteria, but they exit mitosis with an excess of Clb5-dependent kinase activity and cannot relicense DNA molecules for the next round of DNA synthesis, so they should be classified as inviable.

### Using phenotype competitiveness to accelerate the evolutionary algorithm

By ignoring non-competitive phenotypes we can reduce the number of phenotypes that need to be simulated during optimization. To illustrate, we identified the 50 least competitive phenotypes from the $\hat{R}$ values computed from a small set of 100 LH samples. We then generated five subsets of phenotypes to be used in the selection process by eliminating from the full set of 119 phenotypes the 10 least competitive phenotypes, the 20 least competitive phenotypes,..., the 50 least competitive phenotypes. Next, for each subset we performed four independent runs of DE optimization, using always the same initial population of 19 parameter vectors and varying the random numbers used in mutation and crossover operations. Lastly, after 500 generations of DE, we used the final 19 parameter vectors to see how many of the full group of 119 phenotypes are correctly simulated. The results, given in Table 2, show that by simulating only 89 phenotypes, we find parameter vectors that correctly simulate 102–109 of the full set of 119 phenotypes. This success rate is comparable to the results obtained by simulating all 119 phenotypes in every generation of DE. Hence, by ignoring the least competitive phenotypes we can reduce the computational load of the DE algorithm by about 25% in each generation, without losing the ability of the optimization procedure to find sets of parameter values that give the best possible account of all 119 phenotypes. Apparently, we get these 30 least competitive phenotypes for free during the optimization procedure. They are listed in Additional file 1: Table S10.

**Table 2:** Variations in the total number of phenotypes simulated during optimization

**Group**	**# phenotypes in selection**	**# phenotypes captured**
0	119	105–111
1	109	106–108
2	99	104–109
3	89	102–109
4	79	66–105
5	69	58–67

Optimization results (Four individual runs with groups 1–5) with reduced sets of phenotypes.

Of five individual non–greedy DE runs (which produced 105–111 hits out of 119), we found that they all captured 95 of the 119 phenotypes. Of the 24 phenotypes that were missed by at least one of the five DE runs, none were among the 30 least competitive phenotypes identified by the 100 LH samples. Furthermore, three phenotypes (# 67, 74, and 117 listed in Additional file 1: Table S7), all among the four most competitive phenotypes (Additional file 1: Table S10) as identified by 100 LH samples, were consistently missed by the top performing parameter vectors in all five DE runs. These results show that, with a small number of LH samples, we can get an idea of how DE will perform over thousands of samples before convergence. Nonetheless, DE is still necessary to approach an optimal number of hits, since 100 LH samples, in three independent runs, can get at most ∼80 hits. Even with 19,000 LH samples, the maximum number of hits is 92, whereas DE reaches this many hits in ∼25 generations (∼500 samples), indicating that random sampling of parameter space is outperformed by a combination of random sampling (LH) with evolutionary search (DE).

### Order of events

During DE we have applied only the most basic phenotypic constraint on the simulated strains, namely viability or inviability. In our simulations, viability means that cells divide periodically and that cell mass at division converges to a specific value (±5%). Inviability means that the cell mass exceeds 25, which only happens when the cell becomes arrested in the cell cycle, never dividing but continuing to grow. (Other behavior, such as double-period oscillations, was considered neither inviable nor viable).

Additional constraints could be introduced. For example, for a cell to be viable, not only must it divide periodically at a characteristic size, but also it should execute cell cycle events (origin relicensing, origin activation, spindle alignment, Esp1 activation and cell division) in the correct order. And inviable cells should be checked to see that they have arrested in the observed phase of the cell cycle. In addition, we could check other commonly measured cell cycle properties of mutants: for example, cell size at division, and duration of the unbudded phase of the cell cycle. Although we intend to examine these additional constraints in later studies, in this study we have checked the order of events in all 119 strains produced by the optimum parameter vectors. These parameter vectors reproduced the five events for all experimentally viable mutants in the correct order with the following exceptions: 

1. In three viable strains that had no copy of PDS1 (*pds1 Δ, CLB5-db Δ pds1 Δ*, and *clb5 Δ clb6 Δ cdc20 Δ pds1 Δ*), Esp1 was always active ([Esp1] > 0.2), and the model would ideally show that Esp1 degrades cohesins and initiates anaphase separation of sister chromatids before all replicated chromosomes are aligned on the mitotic spindle (a lethal mistake called “mitotic catastrophe”). That such catastrophes are not observed in cells lacking Pds1 (the inhibitor of Esp1) indicates that the model is missing an important control on cohesin degradation. In this case, it is believed that Polo kinase must phosphorylate cohesins before they can be degraded by Esp1, and this effect is not included in the present version of the model. In these *pds1 Δ* mutants, all events other than Esp1 activation took place in the right order; hence, we are justified in considering these mutant strains to be viable.

2. In simulations of three other “viable strains” (*APC-A sic1 Δ cdc6 Δ2-49, CLB2-db Δ multicopy SIC1*, and *APC-A cdh1 Δ multicopy SIC1*) some events did not happen in the right order (e.g., multiple origin-relicensing events in a single cell cycle, no spindle alignment before cell division). On the other hand, there are two experimentally inviable phenotypes (phenotypes 46 and 48: *CLB5-db Δ sic1 Δ* and *GAL-CLB5 sic1 Δ*) for which our parameter vectors exhibit “viability” but the events do not happen in the right order. Hence, these two strains should be correctly classified as inviable.

As a result, even with the event-order constraints, our number of hits is only reduced from 111 to 110. Hence, it appears that by selecting according to our simple definition of viability/inviability, the model (in the majority of cases) automatically reproduces the correct sequence of events in mutant strains. This property of the model is an indication that it is correctly representing the sequence of dependencies in the molecular mechanism underlying cell cycle progression in budding yeast.

### Sensitivity analysis of the model

Sensitivity analysis is widely applied to study biological systems in order to quantify the robustness of biological behavior to changes in model parameters, to determine the most sensitive model parameters and experimental constraints, and to guide further experimental work and model refinement [12]. Our approach to sensitivity analysis, described below, is similar to past studies by Bentele et al. [10].

#### Fragile and robust phenotypes

Our objective function is the number of phenotypes successfully simulated by the model for a particular set of parameter values. In this section, we identify the effects of single parameter perturbations on this function in order to identify those parameters to which the model is most sensitive. When perturbed, these “critical” parameters cause the loss of already captured phenotypes more frequently than non-critical parameters. In addition, because the objective function encompasses all experimental constraints, we can look for links between individual parameters and individual genetic strains. For large and complex networks, such as the budding yeast cell cycle, the identification of such input-output relationships can be challenging and counterintuitive [1]. Nonetheless, we seek such relationships because they can suggest how a control system might be perturbed experimentally in order to achieve particular desired outcomes.

Our approach to sensitivity analysis is to produce a large sample of perturbations away from the best parameter vectors identified by DE. We then ask of this sample which parameters—when perturbed—cause the most drastic loss of correctly simulated phenotypes; these are the critical parameters. The phenotypes most often incorrectly simulated are the fragile phenotypes. To generate the sample of parameter vector perturbations, we must first choose a representative collection of parameter vectors that are most successful in capturing phenotypes. The most successful DE run (non-greedy Run 1, in Figure 7) captured 111 of 119 phenotypes. Of the 19,000 parameter vectors investigated in this run, 3415 defined models that reproduced 111 phenotypes, and of these successful sets we chose 15 at random. Next, we introduce perturbations to each parameter in each of these 15 vectors. In general, each parameter is perturbed ±20%, ±40%, ±60%, ±80% (eight perturbation levels) from its nominal value and in addition each parameter is set to zero (the ninth perturbation level).

Recall that each parameter vector consists of 126 kinetic constants (Additional file 1: Table S1) plus 26 initial conditions (Additional file 1: Table S2). Of the 126 kinetic constants, two were not perturbed: mass doubling time (held constant at 100 min) and fraction of daughter mass acquired from the mother (held constant at 0.4). In addition, the *CLN2* basal expression rate was fixed at zero, since even tiny values of this parameter consistently caused *cln3 Δ bck2 Δ* and *cln3 Δ bck2 Δ sic Δ* strains to be viable, contrary to experimental observations. Also, there were 224 mutant-parameter combinations where setting the parameter to zero did not make sense. For example, for *CKI* overexpression with a *GAL* promoter, it is not sensible to have a zero setting for the synthesis rate of CKI protein. Similarly, for each phenotype, setting the initial cell size to zero is not possible.

Starting from each of the 15 successful parameter vectors chosen from the DE run, we introduced one of the parameter perturbations to create a “new” parameter vector. We then simulated WT cells in glucose (all our parameter vectors at this point define models that reproduce WT viability). Next, we simulated each of the 118 other strains by adjusting the parameter values in the “new” parameter vector according to the rules that mimicked these strains. In total, from the 15 initial parameter vectors we created 20115 new parameter vectors (15 sets × 9 perturbation levels × 149 varying model parameters) and ran 119 simulations per vector. Out of these 2,393,685 simulations, we discarded the ones where setting a parameter to zero was not sensible, leaving us with 2,393,461 to assess model robustness. The total number of hits among the 20115 parameter vectors generated after perturbations in individual parameters ranged from 54 to 111. Although some of the eight phenotypes that were missed by optimization were captured by some of these perturbations, the overall number of hits never exceeded 111. These eight phenotypes (# 12, 46, 48, 55, 66, 67, 74, and 117 listed in Additional file 1: Table S7) were not taken into account in the sensitivity analysis, since our reference point was the outcome of the optimization.

We computed the number of times each of the 111 captured phenotypes was lost after a parameter perturbation, and then we ranked the 111 phenotypes according to their frequency of loss (“fragility”). The 20 most fragile phenotypes (18% of the 111 phenotypes) accounted for 46% of the total number of losses, and each contributed at least 1.5% to this total. Only four of these phenotypes are experimentally inviable, which indicates that viability is more vulnerable to parametric perturbations. On the other hand, out of the 33 single-mutation phenotypes captured before perturbations, only four of them are among the most fragile 20 phenotypes. This prediction aligns with the increasing fragility observed in biological systems with increasing number of structural changes (mutations) [31]. Table 3 shows the 20 most fragile phenotypes. Mutations connected to the Start cyclins (Cln1,2,3) and the G1-stabilizers (Sic1 and Cdh1) are common features of these phenotypes.

**Table 3:** The 20 most fragile phenotypes

**Strain name**	**Phenotype**	**Percent of total losses**
*CLB2-db* Δ multicopy *SIC1*	Viable	3.38
*cln1* Δ *cln2* Δ *cdh1* Δ	Viable	3.19
*CLB2-db* Δ *clb5* Δ *clb6* Δ in galactose	Viable	3.16
*cdc15* Δ *net1-ts cdh1* Δ	Viable	2.85
*GAL-CLB2 cdh1* Δ	Inviable	2.84
*cln1* Δ *cln2* Δ *cdh1* Δ *GAL-CLN2*	Viable	2.74
*CLB2-db* Δ in galactose	Inviable	2.70
*APC-A*	Viable	2.40
*APC-A sic1* Δ	Viable	2.39
*CLB5-db* Δ *pds1* Δ	Viable	2.35
*APC-A cdh1* Δ multicopy *SIC1*	Viable	2.31
*cdh1* Δ	Viable	2.24
*cln1* Δ *cln2* Δ *cln3* Δ *sic* Δ	Viable	2.03
*bck2* Δ	Viable	1.93
*GALL-CDC20 sic1* Δ *cdh1* Δ	Viable	1.84
*APC-A cdh1* Δ in galactose	Inviable	1.72
*clb5* Δ *clb6* Δ *cdc20* Δ *pds1* Δ	Viable	1.69
*cln3* Δ *bck2* Δ *sic* Δ	Inviable	1.67
*cln1* Δ *cln2* Δ	Viable	1.54
*APC-A sic1* Δ *cdc6* Δ2-49	Viable	1.53

These are the phenotypes that are most often lost (i.e., incorrectly simulated) when perturbations are applied to individual model parameters. Fragility decreases from top to bottom.

The 33 most robust phenotypes are all inviable. The first viable phenotype that is also robust is ranked 34^*th*^ on the list, and there are nine other robust viable phenotypes among the top 45 robust phenotypes (see Table 4). The wild type phenotype (in both glucose and galactose growth media) is among the most robust viable phenotypes as expected, since the high number of hits during optimization is strongly dependent on the model’s ability to capture wild type viability. The most common feature of robust-viable genetic strains is the presence of mutations in the EXIT module (*NET1, CDC14, CDC15, PPX, TAB6-1, TEM1*), as compared to the relative fragility of strains with mutations in the START module.

**Table 4:** The 10 most robust viable phenotypes

**Rank**	**Strain name**	**Phenotype**	**Percent of total losses**
34	*TAB6-1 CLB1 clb2* Δ	Viable	0.51
36	*GAL-SIC1*	Viable	0.53
37	*GAL-NET1 GAL-CDC14*	Viable	0.53
39	Multicopy *CDC15*	Viable	0.57
40	*GAL-TEM1*	Viable	0.60
41	WT in glucose	Viable	0.61
42	WT in galactose	Viable	0.62
43	*ppx* Δ	Viable	0.62
44	*TAB6-1*	Viable	0.62
45	*tem1-ts* multicopy *CDC15*	Viable	0.62

These are the viable phenotypes that are least often lost (i.e., incorrectly simulated) when perturbations are applied to individual model parameters. Robustness decreases from top to bottom. *TAB6-1* has lowered efficiency of Cdc14-Net1 complex (RENT) formation compared to WT efficiency.

#### Critical and dispensable parameters

We used the same data set to analyze model robustness with respect to parameters. For each model parameter, we counted the number of times a perturbation caused the loss of a phenotype, and ranked parameters according to their ability to affect our objective function (the total number of captured phenotypes). “Critical” parameters are parameters that when perturbed cause frequent losses of phenotypes. On the other hand, parameters that can be perturbed with little or no change in our objective function are clearly “dispensable” parameters to the optimization process considered here. The 20 most critical parameters, which account for 44% of the total losses, are listed in Table 5. The most critical parameter in our analysis is the total amount of Cdc14, which accords nicely with the experimental result that, among 30 cell cycle genes studied by Moriya et al. [32], the cell cycle was least tolerant to overexpression of *CDC14*.

**Table 5:** The 20 most critical model parameters

**Parameter name**	**Percent of losses**
Total amount of Cdc14	3.14
SPN synthesis rate	2.81
Total amount of Esp1	2.60
Total amount of Net1	2.57
Degradation rate of Cdc20	2.53
PPX inactivation by Esp1	2.51
Efficiency of Cdc14-Net1 complex (RENT) formation	2.50
Time scale for protein activation	2.41
Net1 phosphorylation by Clb2	2.30
Total amount of Mcm1	2.25
Transcriptional activation of *CDC20* by Mcm1	2.17
Transcriptional activation of *CLB2* by Mcm1	2.06
Sigmoidicity of protein activation	1.99
Degradation rate of Swi5	1.99
CKI phosphorylation rate	1.96
Cdh1 inactivation rate	1.83
Total amount of SBF	1.79
Clb2 degradation by active Cdc20	1.76
Polo activation by Clb2	1.67
Synthesis rate of Bck2	1.66

Most critical model parameters that had the largest effects on the objective function upon perturbations. Criticality decreases from top to bottom.

Twelve of the 20 most critical parameters are involved in the EXIT module. Whereas EXIT module parameters are critical in terms of capturing phenotypes, viable strains with mutations in EXIT module genes are highly robust to perturbations when the effects are summed over all parameters in the model. These contrasting results underscore the difference between critical parameters and robust strains. The evaluation of model parameters and phenotypes is performed by looking into different sets of outputs. A robust strain (which is insensitive to changes in a majority of the parameters) is identified by taking perturbations in all parameters into account. On the other hand, a critical parameter (causing the loss of more phenotypes than other parameters once it is perturbed) is identified by taking all phenotypes into account.

We also identified the 70 least critical model parameters, i.e., those parameters with little or no effect on the objective function. To determine if some of these parameters are dispensable as far as our optimization problem is concerned, we constructed a series of “reduced models” by setting more and more of these least-critical parameters to zero. The results, presented in Table 6, demonstrate that we can eliminate the 50 least critical parameters (listed in Additional file 1: Table S11) without seriously degrading the ability of the model to capture at least 105 of the 119 strain phenotypes.

**Table 6:** Performances of the reduced order models

**Reduced model #**	**# Model parameters**	**# Hits**
0	152 (full model)	105–111
1	102	107–107
2	92	93–97
3	82	94–96

Optimization results with the full model and the reduced models (two individual runs for each reduced model). The least sensitive 50 parameters are set to zero in Reduced Model 1, whereas the least sensitive 60 and 70 parameters are set to zero in Reduced Models 2 and 3, respectively.

In an independent repeat of this sensitivity analysis, 49 of the top 50 least critical parameters were the same, and in another repeat after an independent DE run that produced 1803 parameter vectors with 110 hits, 44 of the 50 were the same. It is noteworthy that 25 initial conditions (all except the initial mass) and 6 BUD related parameters always formed the least critical 31 parameters. In hindsight, this is not surprising, because the initial conditions are used only to start up the simulation of a WT cell in glucose. If the WT cell is viable, we replace the “initial” ICs by the values of all variables in a newborn WT cell for all further strain simulations. So the “initial” ICs have no bearing on the calculation of the objective function. Initial mass plays a different role, because if it is too large or too small, then the WT cell may not be correctly simulated. Regarding BUD-related parameters, the BUD variable is part of the model in order to time the appearance of the bud in relation to the onset of DNA synthesis (the ORI variable), but the BUD variable has no effect on further progress through the cell cycle. Hence, the BUD-related parameters have no effect on the viability or inviability of simulated strains. In future versions of the model, where the timing of budding events will enter into the objective function, the BUD-related parameters will no longer be dispensable. Of the remaining 50 least critical parameters (Additional file 1: Table S11), many are “basal” rates of synthesis or degradation and “basal” rates of activation or inactivation. Most of these basal rate constants can be set to zero without seriously degrading the success of the model in capturing viability or inviability of the mutant strains.

#### Strongly connected phenotype-parameter pairs

We also computed the number of times a model parameter, upon perturbation, caused the loss of a specific captured phenotype. The ten most strongly connected mutant-parameter pairs are listed in Table 7. Each of these parameters has changed the phenotype of the corresponding strain for at least 118 of the 135 perturbations (9 perturbation levels × 15 parameter vectors). All of these strains carry multiple mutations, a characteristic of fragile phenotypes. The fragility of these strains is, for the most part, connected to perturbations in mitotic exit parameters. The highly affected strains also tend to carry multiple copies of a wild type gene, suggesting that DE has delicately balanced model parameters to reproduce the viability of these strains.

**Table 7:** Strongly connected phenotype-model parameter pairs

**Phenotype**	**Perturbed model parameter**	**Probability of phenotype loss**
*cdc15* Δ *net1-ts cdh1* Δ	Total amount of Cdc14	1.00
*cdc15* Δ *net1-ts cdh1* Δ	Total amount of Net1	0.99
*CLB2-db* Δ multicopy *SIC1*	Basal SBF dephosphorylation	0.93
*CLB2-db* Δ multicopy *SIC1*	SBF-dependent Cln2 synthesis	0.90
*cln1,2* Δ *cdh1* Δ *GAL-CLN2*	Total amount of Esp1	0.89
*CLB2-db* Δ multicopy *SIC1*	CKI phosphorylation rate	0.88
*cdc15* Δ *net1-ts cdh1* Δ	Efficiency of Cdc14-Net1 complex (RENT) formation	0.88
*cln1,2* Δ *cdh1* Δ *GAL-CLN2*	PPX inactivation by Esp1	0.87
*GALL-CDC20 sic1* Δ *cdh1* Δ	Degradation rate of Cdc20	0.87
*cdc15-ts* multicopy *CDC14*	Total amount of Net1	0.87

Upon a perturbation, with at least 0.87 probability, these parameters caused the loss of the corresponding phenotype (all phenotypes are experimentally viable).

#### Novel phenotypes predicted by the elimination of phosphorylation/dephosphorylation reactions

It is interesting to note that there are only two phosphorylation/dephosphorylation rates among the 20 most critical parameters in Table 5, despite the presence of several more such rates in the model equations. This motivated us to look more carefully into sensitivity analysis results, specifically the perturbations by which parameter values were set to zero. We focused on nine phosphorylation/dephosphorylation rates (a subset of all such rates) that cause inviability in otherwise viable genetic strains as shown in Table 8 (wild type viability was retained when these rates were set to zero). In other words, sensitivity analysis gave us novel phenotypes that highlighted the importance of this class of reactions only under specific mutant backgrounds. For a similar past experimental study that identified Sic1 phosphorylation to be lethal only when one of the Clb-cyclins is deleted, see [33].

**Table 8:** Synthetic lethality induced by elimination of phosphorylation/dephosphorylation reactions

**Eliminated reaction**	**Impacted single mutation strains that are**
	**viable (inviable) before (after) perturbation**
Whi5 phosphorylationby Bck2	*cln3* Δ, Multicopy *BCK2, cdh1* Δ, *sic1* Δ, *swi5* Δ,
	*CLB5-db* Δ, *net1-ts, GAL-CLB2, APC-A*
CKI phosphorylation by Cln2	*bck2 Δ*, *GAL-SIC1, net1-ts, APC-A*
CKI phosphorylation by Clb2	*GAL-CLN3, cdh1* Δ, *GAL-CLB5, CLB1 clb2* Δ
CKI dephosphorylation by Cdc14	*bck2* Δ, *cdh1* Δ, *GAL-CLB2, APC-A*
Whi5 phosphorylation by Cln3	*bck2* Δ, *cdh1* Δ, *APC-A*
SBF phosphorylation by Clb2	*cdh1* Δ, *CLB5-db* Δ, *APC-A*
Whi5 phosphorylation by Cln2	*bck2* Δ,*APC-A*
Whi5 dephosphorylation by Cdc14	*APC-A*
Net1 dephosphorylation by PPX	Multicopy *CDC15*

Upon setting phosphorylation/dephosphorylation rate constants to zero (left column), viability is lost in several single mutation strains (right column).

The three genetic strains that are most commonly affected by elimination of these nine specific phosphorylation/dephosphorylation reactions are *APC-A*, *bck2 Δ* (or Multicopy *BCK2*), and *cdh1 Δ* (Table 8). APC phosphorylation (activation) by Clb2 is set to zero in *APC-A* and this inhibits the degradation of Clb5 and Clb2. Clb5 phosphorylates Whi5 and CKI, and Clb2 phosphorylates SBF and CKI. Hence, increased levels of Clb5 and Clb2 in *APC-A* combined with the elimination of phosphorylation/dephosphorylation reactions upset the net phosphorylation of CKI, Whi5 and SBF that is required for viability. Likewise, Bck2 inhibits CKI and Whi5, and its deletion or overexpression combined with these parametric perturbations cause imbalances between phosphorylation and dephosphorylation rates, leading to inviability. In *cdh1 Δ* background, Clb2 is in excess (as in *APC-A*) since Cdh1 degrades Clb2. Therefore, SBF and CKI phosphorylation is affected the same way as described above for *APC-A*. On the other hand, excess Clb2 in *cdh1 Δ* background leads to higher levels of phosphorylated (inactive) Net1, thereby reducing Net1’s ability to trap Cdc14 in Cdc14-Net1 complex resulting in excess free Cdc14 that impacts net Whi5 phosphorylation. We would like to note that only 31 single mutation strain–parameter pairs, 17.2% of all possible 180 mutant–parameter pairs (20 single mutation strains × 9 phosphorylation/dephosphorylation rates), are affected by the nine phosphorylation/dephosphorylation rates (Table 8). These rare fragilities are identified automatically by the sensitivity analysis, which actually generates hundreds of novel phenotypes starting from the 119 phenotypes we used in optimizing model parameters. Identification of these rare parameter–mutant combinations in such a large input–output space is potentially beneficial to biologists, who are typically faced with the daunting task of performing genetic screens in complex biological networks. Our approach provides an experimental design path to follow with a mathematically simple sampling and optimization framework in the absence of time–series data, but only a set of qualitative observations (phenotypes) available to train the model.

## Conclusions

The physiological characteristics of a living cell—for example, how it progresses through the cell division cycle, or how it responds to external stimuli, or how it develops within a specialized tissue—depend ultimately on the dynamical properties of macromolecular regulatory networks. The dynamics of these networks can be described accurately by systems of differential equations (in a deterministic setting) or sets of reaction probabilities (in a stochastic setting). In principle these systems of equations can be simulated numerically and the results compared to the behavior of living cells under a variety of experimental conditions. Unfortunately this vision of a grand theory of molecular cell biology is subverted by the “curse of parameter space”. Any realistic model of a functional cellular control system will contain dozens of interacting genes, proteins and metabolites and many dozens of rate constants (or reaction probabilities), which are generally unknown. A major part of the challenge of model building in molecular systems biology is to estimate the system parameters from the available experimental data and, in the process, to assess how well the data constrains the model and how well the parameterized model accounts for the data and makes reliable predictions of future experiments. It is in this context that systems biologists need practical approaches to parameter estimation. Brute force exploration of parameter space is not an option. For a model with 100 parameters, even to evaluate parameter vectors at each corner of a hypercube bounding a feasible region of parameter space would take approximately 10^30^ evaluations, a number well beyond foreseeable computational power. In light of this fact, any optimization procedure must propose ways to trade off wider exploration of parameter space for denser sampling of more promising regions found by previous sampling.

Furthermore, the optimization problem is itself a moving target. New experimental observations are continuously being reported, forcing the model to adapt and change. There is no point in employing a heavy duty optimization procedure that provides a single “optimal” solution (at great expense) for a problem that may be outdated tomorrow. What modelers need are computationally light optimization approaches that can help them make quick and flexible progress. The approach we are proposing, based on Latin hypercube sampling and differential evolution, appears well suited to the task. In addition, our approach can be of great use to modelers by highlighting parts of the model where the structure may be insufficient (or overly complex) to explain the observed data.

We believe that our approach is a practical and informative strategy for studying how the assignment of parameter values affects the ability of a complex reaction network to account for extensive data sets. Of course, other approaches are possible, and it is difficult to compare the relative merits and liabilities of various approaches. The only study directly comparable to ours, in the sense of estimating a large number of cell cycle parameters based on mutant phenotype data, is a paper by Panning et al. [34] on global parameter estimation for the Chen-2004 model [16] with a data set consisting of phenotypic characterizations of 115 budding yeast strains. These authors used a combination of two deterministic optimization algorithms: DIRECT (Dividing Rectangles) and MADS (Mesh Adaptive Direct Search). They started with a very good set of parameter values (the Chen-2004 set, which accounts for 103 of the 115 phenotypes) and looked for the global optimum. DIRECT alone improved this result to 107 of 115 phenotypes after 500,000 evaluations of the objective function, most of which were to confirm that the global optimum point did not lie elsewhere. MADS improved the result to 108 very quickly (a few thousand evaluations). At this point, we are not able to provide a direct comparison of our approach with Panning et al., since the two studies used different models and optimized with respect to different phenotypic constraints. However, we plan to include a direct comparison of these two optimization approaches in a later publication.

In conclusion, we have presented a parameter estimation approach for high dimensional ODE models with a large number of experimental constraints. Our approach, which makes use of established parameter sampling and optimization tools, is quite successful in locating points in the parameter space with high model performance, even when the starting point of the search is quite “suboptimal”. Sensitivity analysis of the objective function provides additional information on “critical” and “dispensable” parameters and on fragile and robust phenotypes; information that suggests directions for model refinement. We also used random sampling to measure “competition” between experimental constraints, information that is useful to streamline the number of simulations needed for parameter estimation and to assess trade-offs in a model’s ability to reproduce a given set of experimental observations. Finally, we identified rare parameter–mutant combinations to highlight particular fragilities in the system to illustrate usefulness of our approach to predict novel phenotypes and design new experiments. To conclude, we demonstrated that these methods of parameter estimation and analysis can be a powerful tool to propel systems biology research forward.

## Abbreviations

APC: Anaphase promoting complex; CDK: Cyclin-dependent kinase; DE: Differential evolution; IC: initial conditions; LH: Latin hypercube; ODE: Ordinary differential equation.

## Competing interests

The authors declare that they have no competing interests.

## Authors’ contributions

Conceived and designed the model: CO, TL, KCC, WTB, JJT. Performed the optimization, model reduction and analysis: CO. Wrote the paper: CO, WTB, LTW, JJT. All authors read and approved the final manuscript.

## Supplementary Material

Additional file 1 **Supplementary Tables.** This pdf file includes the supplementary tables referred to in the main text. Click here for file

Additional file 2 **viarray.txt, integrate.cpp, runOPTIMAL.m, runTL.m, integrate.mexw64, OPTIMAL.txt and TLset.txt.** “viarray.txt” holds the viability array of the 119 strains (array values of 1 for viable strains and 2 for inviable strains), “integrate.cpp” is the C subroutine for the ODEs and, integrate.mexw64 is the mex file that allows Matlab to use the C subroutine for solving the ODEs. Altogether, these files reproduce the number of hits with the parameter values from the best performing DE run (OPTIMAL.txt from Additional file 2: Table S4, 111 hits) and the initial parameter values (“initial guess”) before the optimization (TLset.txt from Additional file 2: Table S4, with 72 hits). In order to reproduce these results, Matlab files “runOPTIMAL.m” and “runTL.m” need to be executed, respectively. Click here for file

Additional file 3 **Supplementary Text.** This pdf file includes a detailed description of Latin Hypercube sampling. Click here for file

Additional file 4 **Supplementary Figures.** This pdf file includes two additional figures. Click here for file
