# Supplementary material for: Optimization and model reduction in the high dimensional parameter space of a budding yeast cell cycle model
Source: BMC Syst Biol. 2013 Jun 28;7:53. doi: 10.1186/1752-0509-7-53 (PMC3702416; doi:10.1186/1752-0509-7-53)
Supplement: Additional file 1 — Supplementary Tables. This pdf file includes the supplementary tables referred to in the main text. [file 1752-0509-7-53-S1.pdf]

Table S1: **Model parameters**

Parameter	Description	Parameter	Description
γ	Time scale for protein activation	kd_{swi5}	Degradation rate of Swi5
γ_{ki}	CKI inactivation time scale	$ka_{swi5,14}$	Swi5 activation by Cdc14
γ_{cp}	APC activation time scale	$ki_{swi5,b2}$	Swi5 inactivation by Clb2
γ_{tem}	Tem1 activation time scale	$ka_{m1,b2}$	Mcm1 activation by Clb2
σ	Sigmoidicity of protein activation	ki_{m1}	Basal Mcm1 inactivation
σ_{net}	Sigmoidicity of Net1 activation	ks_{20}	Basal Cdc20 synthesis
ks_{n3}	Cln3 synthesis rate	$ks_{20,m1}$	Mcm1-dependent Cdc20 synthesis
J_{n3}	Michaelis-Menten constant	kd_{20}	Cdc20 degradation
D_{n3}	Dosage of CLN3 gene	ka_{20}	Basal Cdc20 activation
kd_{n3}	Cln3 degradation rate	$kd_{b5,20,i}$	Clb5 degradation by Cdc20
ks_{k2}	Bck2 synthesis rate	$kd_{clb2,20,i}$	Clb2 degradation by Cdc20
kd_{k2}	Bck2 degradation rate	$ki_{20,ori}$	Cdc20 inactivation by spindle checkpoint
kdp_{i5}	Basal Whi5 dephosphorylation	$ka_{cp,b2}$	APC phosphorylation by Clb2
kdp_{i514}	Whi5 dephosphorylation by Cdc14	ki_{cp}	APC inactivation
kp_{i5}	Basal Whi5 phosphorylation	ka_{h1}	Basal Cdh1 activation
kp_{i5n3}	Whi5 phosphorylation by Cln3	$ka_{h1,14}$	Cdh1 activation by Cdc14
kp_{i5k2}	Whi5 phosphorylation by Bck2	ki_{h1}	Basal inactivation of Cdh1
kp_{i5n2}	Whi5 phosphorylation by Cln2	$ki_{h1,e}$	Cdh1 inactivation rate
kp_{i5b5}	Whi5 phosphorylation by Clb5	$e_{h1,n3}$	Cdh1 inactivation by Cln3
kdp_{bf}	Basal SBF dephosphorylation	$e_{h1,n2}$	Cdh1 inactivation by Cln2
kp_{bfb2}	SBF phosphorylation by Clb2	$e_{h1,b5}$	Cdh1 inactivation by Clb5
ks_{n2}	Basal Cln2 synthesis rate	$e_{h1,b2}$	Cdh1 inactivation by Clb2
ks_{n2bf}	SBF-dependent Cln2 synthesis	kdp_{net}	Basal Net1 dephosphorylation
kd_{n2}	Cln2 degradation	$kdp_{net,14}$	Net1 dephosphorylation by Cdc14
ks_{ki}	Basal CKI synthesis rate	$kdp_{net,px}$	Net1 dephosphorylation by PPX
$ks_{ki,swi5}$	Swi5-dependent CKI synthesis	kp_{net}	Basal Net1 phosphorylation
kd_{ki}	Degradation rate of CKI	$kp_{net,b2}$	Net1 phosphorylation by Clb2
kd_{kip}	Degradation rate of CKIP	$kp_{net,en}$	Net1 phosphorylation by MEN
$kp_{ki,e}$	CKI phosphorylation rate	$kp_{net,15}$	Net1 phosphorylation by Cdc15
$e_{ki,n3}$	CKI phosphorylation by Cln3	ka_{px}	Basal PPX activation
$e_{ki,k2}$	CKI phosphorylation by Bck2	ki_{px}	Basal PPX inactivation
$e_{ki,n2}$	CKI phosphorylation by Cln2	$ki_{px,p1}$	PPX inactivation by Esp1
$e_{ki,b5}$	CKI phosphorylation by Clb5	ks_{pds}	Basal Pds1 synthesis
$e_{ki,b2}$	CKI phosphorylation by Clb2	kd_{pds}	Basal Pds1 degradation
kdp_{ki}	Basal dephosphorylation of CKI	$kd_{pds,20}$	Pds1 degradation by Cdc20A
$kdp_{ki,14}$	CKI dephosphorylation by Cdc14	$kd_{pds,20,i}$	Pds1 degradation by Cdc20
ks_{b5}	Basal Clb5 synthesis	ka_{15}	Basal Cdc15 activation
$ks_{b5,bf}$	SBF-dependent Clb5 synthesis	$ka_{15,14}$	Cdc15 activation by Cdc14
kd_{b5}	Basal Clb5 degradation	ki_{15}	Basal Cdc15 inactivation
$kd_{b5,20}$	Clb5 degradation by Cdc20A	$ki_{15,b2}$	Cdc15 inactivation by Clb2
ks_{b2}	Basal Clb2 synthesis	ka_{tem}	Basal Tem1 activation
$ks_{b2,m1}$	Mcm1-dependent Clb2 synthesis	$ka_{tem,lo}$	Tem1 activation by Polo
kd_{b2}	Basal Clb2 degradation	$ka_{tem,p1}$	Tem1 activation by Esp1
$kd_{b2,20}$	Clb2 degradation by Cdc20A	ki_{tem}	Basal inactivation of Tem1
$kd_{b2,h1}$	Clb2 degradation by Cdh1A	$ki_{tem,px}$	Tem1 inactivation by PPX

Continued on next page

Table S1 – *Continued from previous page*

Parameter	Description	Parameter	Description
$ks_{bud,e}$	Time scale for BUD synthesis	ks_{lo}	Basal Polo synthesis
$e_{bud,n3}$	Cln3 activation of BUD	$ks_{lo,m1}$	Mcm1-dependent synthesis of Polo
$e_{bud,n2}$	Cln2 activation of BUD	kd_{lo}	Basal Polo degradation
$e_{bud,b5}$	Clb5 activation of BUD	$kd_{lo,h1}$	Polo degradation by Cdh1
$e_{bud,b2}$	Clb2 activation of BUD	ka_{lo}	Basal Polo activation
kd_{bud}	BUD degradation	$ka_{lo,b2}$	Polo activation by Clb2
ks_{spn}	SPN synthesis	ki_{lo}	Basal Polo inactivation
kd_{spn}	SPN degradation	kas_{net}	Efficiency of Cdc14-Net1 complex (RENT) formation
J_{spn}	SPN synthesis threshold	f	Fraction of mass retained by daughter at division
$ks_{ori,e}$	Time scale for ORI synthesis	MDT	Mass doubling time
$e_{ori,b5}$	Clb5 activation of ORI	$Whi5_T$	Total Whi5
$e_{ori,b2}$	Clb2 activation of ORI	SBF_T	Total SBF
kd_{ori}	Degradation of ORI	$Mcm1_T$	Total Mcm1
ks_{swi5}	Basal Swi5 synthesis	APC_T	Total APC
$ks_{swi5,m1}$	Mcm1-dependent Swi5 synthesis	$Cdh1_T$	Total Cdh1
		$Net1_T$	Total Net1
		$Cdc14_T$	Total Cdc14
		PPX_T	Total PPX
		$Esp1_T$	Total Esp1
		$Cdc15_T$	Total Cdc15
		$Tem1_T$	Total Tem1

Table S2: **Model variables**

Variable	Description
V	Cell size
[Cln3]	Concentration of G1 cyclin Cln3
[Bck2]	Concentration of Bck2, an activator for START transition
[WHI5dep]	Dephosphorylated (active) Whi5
[SBFdep]	Dephosphorylated SBF
[Cln2]	Total concentration of G1 cyclins Cln1,2
[CKI_T]	Total concentration of Sic1+Cdc6, stoichiometric inhibitors of Clb5,6 and Clb1,2
[CKI_P]	Phosphorylated form of Sic1+Cdc6, stoichiometric inhibitors of Clb5,6 and Clb1,2
[$Clb5_T$]	Total concentration of free B cyclins Clb5,6
[$Clb2_T$]	Total concentration of free B cyclins Clb1,2
[BUD]	Variable for bud emergence progression
[ORI]	Variable for DNA synthesis progression
[SPN]	Variable for spindle assembly progression
[$Swi5_T$]	Total concentration of SWI5, transcription factor for CKI synthesis
[$CDC20_T$]	Total concentration of Cdc20, a protein involved in Clb5,6, Clb1,2 and Pds1 degradation
[APCP]	Phosphorylated (active) APC, activates Cdc20 by complex formation
[$Cdh1_A$]	Active Cdh1 degrading Clb1,2 and Polo kinase
[Net1dep]	Dephosphorylated (active) Net1, a stoichiometric inhibitor of Cdc14
[PPX]	Phosphatase that dephosphorylates Net1
[$Pds1_T$]	Securin, Esp1's stoichiometric inhibitor
[Cdc15]	Kinase, when complexed with Tem1, phosphorylates Net1
[Tem1]	Protein, when complexed with Cdc15, phosphorylates Net1
[$Polo_T$]	Total concentration of Polo kinase that activates Tem1
[$Polo_A$]	Concentration of phosphorylated (active) Polo kinase that activates Tem1
[Cdc20A-APC]	Complex formed from active Cdc20 and APC
[Cdc20A-APCP]	Complex formed from active Cdc20 and APC-P

Initial values of these variables in the wild type (glucose) simulations are also optimized with the model parameters. Each variable corresponds to a single ODE.

Table S3: **Initial values ("TL set"), optimal values and optimal ranges of model parameters**

Parameter	Initial value	Optimal value (range)	Parameter	Initial value	Optimal value (range)
γ	1	0.4902 (0.4560–0.4925)	kd_{swi5}	0.08	0.1017 (0.1012–0.1027)
γ_{ki}	10	10.4240 (4.1816–18.9217)	$ka_{swi5,14}$	2	1.4203 (0.5683–2.5625)
γ_{cp}	1	0.5495 (0.5249–0.5576)	$ki_{swi5,b2}$	0.05	0.0456 (0–0.0820)
γ_{tem}	1	0.6599 (0.5258–0.9239)	$ka_{m1,b2}$	10	11.1777 (11.1736–13.4084)
σ	10	9.9996 (9.9596–10.2929)	ki_{m1}	1	1.2121 (1.1765–1.3243)
σ_{net}	10	11.4240 (9.1481–20.6561)	ks_{20}	0.006	0.0087 (0.0067–0.0155)
ks_{n3}	1.5	0.8110 (0.8104–0.8125)	$ks_{20,m1}$	0.6	0.6643 (0.6606–0.6662)
J_{n3}	6	5.3729 (5.3398–5.3947)	kd_{20}	0.3	0.2687 (0.2677–0.2700)
D_{n3}	1	0.6792 (0.6775–0.7879)	ka_{20}	0.1	0.0816 (0.0651–0.0980)
kd_{n3}	3	3.0713 (3.0653–3.0762)	$kd_{b5,20,i}$	0.012	0.0161 (0.0064–0.0257)
ks_{k2}	0.1350	0.1651 (0.1649–0.1652)	$kd_{clb2,20,i}$	0.032	0.0323 (0.0321–0.0323)
kd_{k2}	2.5	2.3281 (2.3133–2.3372)	$ki_{20,ori}$	8	8.0846 (1.6034–14.5784)
kdp_{i5}	1	0.6832 (0.6727–0.6842)	$ka_{cp,b2}$	1	1.3485 (1.3469–1.3928)
kdp_{i514}	0.1	0.1429 (0.0856–0.2576)	ki_{cp}	1	1.0923 (1.0899–1.0926)
kp_{i5}	0.1	0.1145 (0.1083–0.1157)	ka_{h1}	1	1.0709 (1.0577–1.2707)
kp_{i5n3}	6	8.0298 (8.0204–8.0333)	$ka_{h1,14}$	7.5	6.8649 (6.8336–6.8679)
kp_{i5k2}	6	6.0771 (6.0205–6.0881)	ki_{h1}	0.1	0.1284 (0–0.2317)
kp_{i5n2}	15	10.2359 (1.9094–15.4576)	$ki_{h1,e}$	1	1.3698 (1.3696–1.3702)
kp_{i5b5}	0.1	0.0628 (0–00.1137)	$e_{h1,n3}$	0.25	0.3868 (0–0.7687)
kdp_{bf}	1	0.8169 (0.6551–0.8227)	$e_{h1,n2}$	0.7	0.6046 (0.4828–0.8434)
kp_{bfb2}	8	9.2818 (9.2483–11.1022)	$e_{h1,b5}$	7	7.1387 (5.7060–8.7046)
ks_{n2}	0	0 (fixed)	$e_{h1,b2}$	8	6.7021 (6.6694–6.7065)
ks_{n2bf}	0.5	0.5141 (0.5129–0.5336)	kdp_{net}	1	0.7886 (0.7766–0.8026)
kd_{n2}	0.25	0.3394 (0.3391–0.4755)	$kdp_{net,14}$	0.1	0.0672 (0–0.1074)
ks_{ki}	0.0120	0.0082 (0.0081–0.0097)	$kdp_{net,px}$	10	9.9244 (8.4678–12.7017)
$ks_{ki,swi5}$	0.12	0.0962 (0.0910–0.0962)	kp_{net}	0.1	0.1327 (0.0265–0.2123)
kd_{ki}	0.01	0.0155 (0.0154–0.0155)	$kp_{net,b2}$	1	0.9967 (0.9958–1.0238)
kd_{kip}	2	1.6428 (1.3143–2.2999)	$kp_{net,en}$	10	8.1639 (3.2568–14.9003)
$kp_{ki,e}$	1	1.0393 (1.0276–1.0408)	$kp_{net,15}$	0.05	0.0601 (0.0240–0.1082)
$e_{ki,n3}$	2.5	2.1528 (0-3.0160)	ka_{px}	1	0.6471 (0.6467–0.7798)
$e_{ki,k2}$	0.5	0.7061 (0-1.0993)	ki_{px}	0.1	0.1289 (0–0.2386)
$e_{ki,n2}$	1	1.0313 (0.8247–1.0330)	$ki_{px,p1}$	3	3.3649 (3.3577–4.0812)
$e_{ki,b5}$	3	2.3303 (0.9314–2.8124)	ks_{pds}	0.03	0.0306 (0.0245–0.0427)
$e_{ki,b2}$	4	2.0135 (1.6187–2.0234)	kd_{pds}	0.05	0.0355 (0.0071–0.0638)
kdp_{ki}	1	0.8285 (0.8149–0.8285)	$kd_{pds,20}$	3	2.4469 (1.4687–3.9250)
$kdp_{ki,14}$	7	3.2185 (2.7115–3.8867)	$kd_{pds,20,i}$	0.3	0.3639 (0.2895–0.6581)
ks_{b5}	0.0016	0.0024 (0.0019–0.0024)	ka_{15}	0.1	0.0858 (0–0.1547)
$ks_{b5,bf}$	0.01	0.0110 (0.0087–0.0131)	$ka_{15,14}$	5	4.5132 (2.7229–8.1686)
kd_{b5}	0.01	0.0053 (0.0052–0.0076)	ki_{15}	1	0.7061 (0.2770–1.1839)
$kd_{b5,20}$	0.16	0.1488 (0.1479–0.1788)	$ki_{15,b2}$	1	0.6324 (0–1.2013)
ks_{b2}	0.0020	0.0029 (0.0029–0.0029)	ka_{tem}	0.1	0.1156 (0–0.2089)
$ks_{b2,m1}$	0.15	0.1039 (0.1037-0.1041)	$ka_{tem,lo}$	2	2.4371 (1.4611–4.3845)
kd_{b2}	0.003	0.0024 (0.0014–0.0028)	$ka_{tem,p1}$	0.1	0.1158 (0–0.2212)

Continued on next page

Table S3 – *Continued from previous page*

Parameter	Initial value	Optimal value (range)	Parameter	Initial value	Optimal value (range)
$kd_{b2,20}$	0.09	0.1215 (0.1215–0.1219)	ki_{tem}	1	0.8710 (0.6953–1.5641)
$kd_{b2,h1}$	0.4	0.5225 (0.4185–0.5243)	$ki_{tem,px}$	2	1.9067 (0–3.6236)
$ks_{bud,e}$	0.2	0.2073 (0-0.3733)	ks_{lo}	0.01	0.0110 (0–0.0207)
$e_{bud,n3}$	0.05	0.0502 (0-0.0910)	$ks_{lo,m1}$	0.1	0.1023 (0.0413–0.1938)
$e_{bud,n2}$	0.25	0.3263 (0-0.5888)	kd_{lo}	0.01	0.0133 (0–0.0233)
$e_{bud,b5}$	1	0.8492 (0-1.5286)	$kd_{lo,h1}$	0.03	0.0377 (0–0.0678)
$e_{bud,b2}$	0.1	0.0597 (0-0.1097)	ka_{lo}	0.1	0.0644 (0–0.1158)
kd_{bud}	0.06	0.0868 (0–0.1575)	$ka_{lo,b2}$	3	3.0066 (2.9704–3.1893)
ks_{spn}	0.1	0.1399 (0.1385–0.1413)	ki_{lo}	1	1.6499 (1.5454–1.8544)
kd_{spn}	0.06	0.0584 (0.0470–0.0700)	kas_{net}	1	1.2550 (1.2516–1.2579)
J_{spn}	0.14	0.1041 (0.1016–0.1251)	f	0.4	0.4 (fixed)
$ks_{ori,e}$	2	2.4029 (1.4357–3.3643)	MDT	100	100 (fixed)
$e_{ori,b5}$	0.9	0.7772 (0.4596–1.4075)	$Whi5_T$	3	2.4731 (2.4319–2.4831)
$e_{ori,b2}$	0.45	0.5988 (0.2395–0.9579)	SBF_T	1	1.0882 (1.0771–1.0971)
kd_{ori}	0.06	0.0502 (0-0.0904)	$Mcm1_T$	1	0.7003 (0.6931–0.7046)
ks_{swi5}	0.0050	0.0045 (0.0027–0.0063)	APC_T	25	34.8950 (27.8941–41.9203)
$ks_{swi5,m1}$	0.08	0.1029 (0.1022–0.1030)	$Cdh1_T$	1	1.2356 (1.2330–1.2440)
			$Net1_T$	3	3.0336 (2.9878–3.0344)
			$Cdc14_T$	2	2.0512 (2.0441–2.0530)
			PPX_T	1	1.1085 (0.8870–1.5520)
			$Esp1_T$	0.5	0.3851 (0.3850–0.3858)
			Cdc15_T	1	0.8228 (0.3292–1.4830)
			$Tem1_T$	1	1.4117 (0.8446–2.6806)

Initial values are from the “TL set” described in the main text. Optimal values come from averaging 3415 sets of parameter values that captured 111 phenotypes in the most optimal DE run, whereas the optimal ranges are computed from the 8158 sets of parameter values (that also capture the same 111 phenotypes) generated during sensitivity analysis.

Table S4: **Initial values ("TL set"), optimal values and optimal ranges of initial conditions**

Variable	Initial value	Optimal value (range)
V_0	1.25	1.0809 (0.1783–2.3025)
$[Cln3]_0$	0.18	0.2545 (0–0.4590)
$[Bck2]_0$	0.066	0.0855 (0–0.1556)
$[WHI5dep]_0$	2.02	3.2035 (0–6.2711)
$[SBFdep]_0$	0.99	1.0392 (0–1.8962)
$[Cln2]_0$	0.1	0.1198 (0–0.2219)
$[CKI_T]_0$	1.05	1.2733 (0–2.5036)
$[CKI_P]_0$	0.1	0.1089 (0–0.1979)
$[Clb5_T]_0$	0.29	0.2875 (0–0.5203)
$[Clb2_T]_0$	0.1	0.1297 (0–0.2334)
$[BUD]_0$	0.05	0.0226 (0–0.0407)
$[ORI]_0$	0.1	0.0865 (0–0.1800)
$[SPN]_0$	0.11	0.1057 (0–0.1929)
$[Swi5_T]_0$	0.21	0.1841 (0–0.3416)
$[CDC20_T]_0$	0.02	0.0214 (0–0.0387)
$[APCP]_0$	0.1	0.0822 (0–0.1794)
$[Cdh1_A]_0$	1	1.0981 (0–1.9855)
$[Net1dep]_0$	2.7	2.4698 (0–4.4491)
$[PPX]_0$	0.87	1.0024 (0–1.8045)
$[Pds1_T]_0$	0.24	0.2686 (0–0.4877)
$[Cdc15]_0$	0.1	0.1044 (0–0.1967)
$[Tem1]_0$	0.1	0.0922 (0–0.1836)
$[Polo_T]_0$	0.67	0.9371 (0–1.7368)
$[Polo_A]_0$	0.1	0.0884 (0–0.1591)
$[Cdc20A\text{-}APC]_0$	0.1	0.1121 (0–0.2020)
$[Cdc20A\text{-}APCP]_0$	0.1	0.0922 (0–0.1667)

Initial values are referred to as the "TL set" in the main text. Optimal values come from averaging 3415 sets of parameter values that captured 111 phenotypes in the most optimal DE run, whereas the optimal ranges originate from the 8158 sets of parameter values (that also capture the same 111 phenotypes) generated during sensitivity analysis.

Table S5: **List of 119 phenotypes**

Phenotype #	Phenotype name	Viable/Inviable	Changes in WT parameters
1	WT in glucose	Viable	NONE
2	WT in galactose	Viable	MDT=150; f=0.48;
3	*cln3*Δ	Viable	D_{n3}=0; [CLN3]=0;
4	*bck2*Δ	Viable	ks_{k2}=0; [BCK2]=0;
5	*cln3*Δ *bck2*Δ	Inviable	D_{n3}=0; [CLN3]=0; ks_{k2}=0;[BCK2]=0;
6	*cln3*Δ *bck2*Δ multicopy *CLN2*	Inviable	D_{n3}=0; [CLN3]=0; ks_{k2}=0; [BCK2]=0; ks_{n2bf}=ks_{n2bf}*2;
7	*cln3*Δ *bck2*Δ *sic*Δ	Inviable	D_{n3}=0; [CLN3]=0; ks_{k2}=0; [BCK2]=0; ks_{ki}=ks_{ki}*0.125; $ks_{ki,swi5}$=$ks_{ki,swi5}$*0.125; [CKIT]=0.2;
8	*cln3*Δ *bck2*Δ *whi5*Δ	Viable	D_{n3}=0; [CLN3]=0; ks_{k2}=0; [BCK2]=0; WHI5T=0; [WHI5A]=0;
9	*GAL-CLN3*	Viable	MDT=150; f=0.48; D_{n3}=D_{n3}*20;
10	Multicopy *BCK2*	Viable	ks_{k2}=ks_{k2}*17;
11	*cln1*Δ *cln2*Δ	Viable	ks_{n2}=0; ks_{n2bf}=0; [CLN2]=0;
12	*cln1*Δ *cln2*Δ *bck2*Δ	Viable	ks_{n2}=0; ks_{n2bf}=0; [CLN2]=0; ks_{k2}=0; [BCK2]=0;
13	*cln1*Δ *cln2*Δ *sic1*Δ	Viable	ks_{n2}=0; ks_{n2bf}=0; [CLN2]=0; ks_{ki}=ks_{ki}*0.125; $ks_{ki,swi5}$=$ks_{ki,swi5}$*0.125; [CKIT]=0.2;
14	*cln1*Δ *cln2*Δ *cki*Δ	Viable	ks_{n2}=0; ks_{n2bf}=0; [CLN2]=0; ks_{ki}=0; $ks_{ki,swi5}$=0; [CKIT]=0; [CKIP]=0;
15	*cln1*Δ *cln2*Δ *GAL-SIC1*	Inviable	ks_{n2}=0; ks_{n2bf}=0; [CLN2]=0; MDT=150; f=0.48; ks_{ki}=ks_{ki}*33.33;
16	*cln1*Δ *cln2*Δ *GAL-CLN2*	Viable	ks_{n2bf}=0; MDT=150; f=0.48; ks_{n2}=0.15;
17	*cln1*Δ *cln2*Δ *GAL-SIC1 GAL-CLN2*	Viable	ks_{n2bf}=0; MDT=150; f=0.48; ks_{n2}=0.15; ks_{ki}=ks_{ki}*33.33;
18	*cln1*Δ *cln2*Δ *cdh1*Δ	Viable	ks_{n2}=0; ks_{n2bf}=0; [CLN2]=0; CDH1T=0; [CDH1A]=0;
19	*cln1*Δ *cln2*Δ *cdh1*Δ *GAL-SIC1*	Inviable	ks_{n2}=0; ks_{n2bf}=0; [CLN2]=0; CDH1T=0; [CDH1A]=0; MDT=150; f=0.48; ks_{ki}=ks_{ki}*33.33;
20	*cln1*Δ *cln2*Δ *cdh1*Δ *GAL-CLN2*	Viable	ks_{n2bf}=0; CDH1T=0; [CDH1A]=0; MDT=150; f=0.48; ks_{n2}=0.15;
21	*cln1*Δ *cln2*Δ *cdh1*Δ *GAL-SIC1 GAL-CLN2*	Viable	ks_{n2bf}=0; CDH1T=0; [CDH1A]=0; MDT=150; f=0.48; ks_{n2}=0.15; ks_{ki}=ks_{ki}*33.33;
22	*cln1*Δ *cln2*Δ *cln3*Δ	Inviable	ks_{n2}=0; ks_{n2bf}=0; [CLN2]=0; D_{n3}=0; [CLN3]=0;
23	*cln1*Δ *cln2*Δ *cln3*Δ *GAL-CLN2*	Viable	ks_{n2bf}=0; D_{n3}=0; [CLN3]=0; MDT=150; f=0.48; ks_{n2}=0.15;

Continued on next page

Phenotype #	Phenotype name	Viable/Inviable	Changes in WT parameters
24	*cln1Δ cln2Δ cln3Δ GAL-CLN3*	Viable	ks_{n2}=0; ks_{n2bf}=0; [CLN2]=0;<br>MDT=150; f=0.48; D_{n3}=D_{n3}*20;
25	*cln1Δ cln2Δ cln3Δ sicΔ*	Viable	ks_{n2}=0; ks_{n2bf}=0; [CLN2]=0;<br>D_{n3}=0; [CLN3]=0;<br>ks_{ki}=ks_{ki}*0.125; $ks_{ki,swi5}$=$ks_{ki,swi5}$*0.125;<br>[CKIT]=0.2;
26	*cln1Δ cln2Δ cln3Δ* multicopy *BCK2*	Viable	ks_{n2}=0; ks_{n2bf}=0; [CLN2]=0;<br>D_{n3}=0; [CLN3]=0;<br>ks_{k2}=ks_{k2}*17;
27	*cln1Δ cln2Δ cln3Δ bck2Δ GAL-CLN2*	Viable	ks_{n2bf}=0;<br>D_{n3}=0; [CLN3]=0;<br>ks_{k2}=0; [BCK2]=0;<br>MDT=150; f=0.48; ks_{n2}=0.15;
28	*cln1Δ cln2Δ cln3Δ* multicopy *CLB5*	Viable	ks_{n2}=0; ks_{n2bf}=0; [CLN2]=0;<br>D_{n3}=0; [CLN3]=0;<br>ks_{b5}=ks_{b5}=*5.33; $ks_{b5,bf}$=$ks_{b5,bf}$*5.33;
29	*cln1Δ cln2Δ cln3Δ GAL-CLB5*	Viable	ks_{n2}=0; ks_{n2bf}=0; [CLN2]=0;<br>D_{n3}=0; [CLN3]=0;<br>MDT=150; f=0.48; ks_{b5}=0.04;
30	*cln1Δ cln2Δ cln3Δ GAL-CLB2*	Inviable	ks_{n2}=0; ks_{n2bf}=0; [CLN2]=0;<br>D_{n3}=0; [CLN3]=0;<br>MDT=150; f=0.48; ks_{b2}=ks_{b2}*49.40;
31	*cln1Δ cln2Δ cln3Δ cdh1Δ*	Inviable	ks_{n2}=0; ks_{n2bf}=0; [CLN2]=0;<br>D_{n3}=0; [CLN3]=0;<br>CDH1T=0; [CDH1A]=0;
32	*cln2Δ cln3Δ apc-ts*	Inviable	ks_{n2}=0; ks_{n2bf}=0; [CLN2]=0;<br>D_{n3}=0; [CLN3]=0;<br>ks_{20}=0; $ks_{20,m1}$=0; CDH1T=0; [CDH1A]=0;
33	*cdh1Δ*	Viable	CDH1T=0; [CDH1A]=0;
34	*CDH1* constitutively active	Inviable	CDH1T=CDH1T*3; $ki_{h1,e}$=0;
35	*sic1Δ*	Viable	ks_{ki}=ks_{ki}*0.125; $ks_{ki,swi5}$=$ks_{ki,swi5}$*0.125;<br>[CKIT]=0.2;
36	*GAL-SIC1*	Viable	MDT=150; f=0.48; ks_{ki}=ks_{ki}*33.33;
37	*GAL-SIC1-dbΔ*	Inviable	MDT=150; f=0.48; ks_{ki}=ks_{ki}*33.33; kd_{kip}=0;
38	*sic1Δ cdc6Δ (ckiΔ)*	Viable	ks_{ki}=0; $ks_{ki,swi5}$=0; [CKIT]=0; [CKIP]=0;
39	*swi5Δ*	Viable	ks_{swi5}=0; $ks_{swi5,m1}$=0; [SWI5T]=0;
40	*sic1Δ cdc6Δ2-49 cdh1Δ*	Inviable	ks_{ki}=0; $ks_{ki,swi5}$=0; [CKIT]=0; [CKIP]=0;<br>CDH1T=0; [CDH1A]=0;
41	*swi5Δ cdh1Δ*	Inviable	ks_{swi5}=0; $ks_{swi5,m1}$=0; [SWI5T]=0;<br>CDH1T=0; [CDH1A]=0;
42	*swi5Δ cdh1Δ GAL-SIC1*	Viable	ks_{swi5}=0; $ks_{swi5,m1}$=0; [SWI5T]=0;<br>CDH1T=0; [CDH1A]=0;<br>MDT=150; f=0.48; ks_{ki}=ks_{ki}*33.33;
43	*clb5Δ clb6Δ*	Viable	ks_{b5}=0; $ks_{b5,bf}$=0; [CLB5T]=0;
44	*clb5Δ clb6Δ cln1Δ cln2Δ*	Inviable	ks_{b5}=0; $ks_{b5,bf}$=0; [CLB5T]=0;<br>ks_{n2}=0; ks_{n2bf}=0; [CLN2]=0;
45	*CLB5-dbΔ*	Viable	$kd_{b5,20}$=0; $kd_{b5,20,i}$=0;

Continued on next page

Table S5 – *Continued from previous page*

Phenotype #	Phenotype name	Viable/Inviable	Changes in WT parameters
46	*CLB5-db*Δ *sic1*Δ	Inviable	$kd_{b5,20}$=0; $kd_{b5,20,i}$=0; ks_{ki}=ks_{ki}*0.125; $ks_{ki,swi5}$=$ks_{ki,swi5}$*0.125; [CKIT]=0.2;
47	*GAL-CLB5*	Viable	MDT=150; f=0.48; ks_{b5}=0.04;
48	*GAL-CLB5 sic1*Δ	Inviable	MDT=150; f=0.48; ks_{b5}=0.04; ks_{ki}=ks_{ki}*0.125; $ks_{ki,swi5}$=$ks_{ki,swi5}$*0.125; [CKIT]=0.2;
49	*GAL-CLB5 cdh1*Δ	Inviable	MDT=150; f=0.48; ks_{b5}=0.04; CDH1T=0; [CDH1A]=0;
50	*GAL-CLB5-db*Δ	Inviable	MDT=150; f=0.48; ks_{b5}=0.04; $kd_{b5,20}$=0; $kd_{b5,20,i}$=0;
51	*clb1*Δ *clb2*Δ	Inviable	ks_{b2}=0; $ks_{b2,m1}$=0; [CLB2T]=0;
52	*clb1*Δ *clb2*Δ *clb5*Δ *clb6*Δ	Inviable	ks_{b2}=0; $ks_{b2,m1}$=0; [CLB2T]=0; ks_{b5}=0; $ks_{b5,bf}$=0; [CLB5T]=0;
53	*GAL-CLB2*	Viable	MDT=150; f=0.48; ks_{b2}=ks_{b2}*49.40;
54	Multicopy *GAL-CLB2*	Inviable	MDT=150; f=0.48; ks_{b2}=ks_{b2}*242.42;
55	*GAL-CLB2 sic1*Δ	Inviable	MDT=150; f=0.48; ks_{b2}=ks_{b2}*49.40; ks_{ki}=ks_{ki}*0.125; $ks_{ki,swi5}$=$ks_{ki,swi5}$*0.125; [CKIT]=0.2;
56	*GAL-CLB2 cdh1*Δ	Inviable	MDT=150; f=0.48; ks_{b2}=ks_{b2}*49.40; CDH1T=0; [CDH1A]=0;
57	*GAL-CLB2 swi5*Δ	Inviable	MDT=150; f=0.48; ks_{b2}=ks_{b2}*49.40; ks_{swi5}=0; $ks_{swi5,m1}$=0; [SWI5T]=0;
58	*CLB2-db*Δ	Inviable	$kd_{b2,20}$=0; $kd_{b2,h1}$=$kd_{b2,h1}$*0.09; $kd_{b2,20,i}$=0;
59	*CLB2-db*Δ in galactose	Inviable	$kd_{b2,20}$=0; $kd_{b2,h1}$=$kd_{b2,h1}$*0.09; $kd_{b2,20,i}$=0; MDT=150; f=0.48;
60	*CLB2-db*Δ *GAL-SIC1*	Viable	$kd_{b2,20}$=0; $kd_{b2,h1}$=$kd_{b2,h1}$*0.09; $kd_{b2,20,i}$=0; MDT=150; f=0.48; ks_{ki}=ks_{ki}*33.33;
61	*CLB2-db*Δ multicopy *SIC1*	Viable	$kd_{b2,20}$=0; $kd_{b2,h1}$=$kd_{b2,h1}$*0.09; $kd_{b2,20,i}$=0; ks_{ki}=ks_{ki}*65; $ks_{ki,swi5}$=$ks_{ki,swi5}$*65;
62	*CLB2-db*Δ *clb5*Δ *clb6*Δ	Inviable	$kd_{b2,20}$=0; $kd_{b2,h1}$=$kd_{b2,h1}$*0.09; $kd_{b2,20,i}$=0; ks_{b5}=0; $ks_{b5,bf}$=0; [CLB5T]=0;
63	*CLB2-db*Δ *clb5*Δ *clb6*Δ in galactose	Viable	$kd_{b2,20}$=0; $kd_{b2,h1}$=$kd_{b2,h1}$*0.09; $kd_{b2,20,i}$=0; ks_{b5}=0; $ks_{b5,bf}$=0; [CLB5T]=0; MDT=150; f=0.48;
64	*GAL-CLB2-db*Δ	Inviable	$kd_{b2,20}$=0; $kd_{b2,h1}$=$kd_{b2,h1}$*0.09; $kd_{b2,20,i}$=0; MDT=150; f=0.48; ks_{b2}=ks_{b2}*49.40;
65	*CLB1 clb2*Δ	Viable	ks_{b2}=ks_{b2}*0.33; $ks_{b2,m1}$=$ks_{b2,m1}$*0.33;
66	*CLB1 clb2*Δ *cdh1*Δ	Inviable	ks_{b2}=ks_{b2}*0.33; $ks_{b2,m1}$=$ks_{b2,m1}$*0.33; CDH1T=0; [CDH1A]=0;
67	*CLB1 clb2*Δ *pds1*Δ	Inviable	ks_{b2}=ks_{b2}*0.33; $ks_{b2,m1}$=$ks_{b2,m1}$*0.33; ks_{pds}=0; [PDS1T]=0;
68	*cdc20-ts* (*cdc20*Δ)	Inviable	ks_{20}=0; $ks_{20,m1}$=0; [CDC20T]=0; [CDC20A-APCP]=0;
69	*clb5*Δ *clb6*Δ *cdc20*Δ	Inviable	ks_{b5}=0; $ks_{b5,bf}$=0; [CLB5T]=0; ks_{20}=0; $ks_{20,m1}$=0; [CDC20T]=0; [CDC20A-APCP]=0;
70	*cdc20*Δ *pds1*Δ	Inviable	ks_{20}=0; $ks_{20,m1}$=0; [CDC20T]=0; [CDC20A-APCP]=0; ks_{pds}=0; [PDS1T]=0;
71	*clb5*Δ *clb6*Δ *cdc20*Δ *pds1*Δ	Viable	ks_{b5}=0; $ks_{b5,bf}$=0; [CLB5T]=0;

Continued on next page

Table S5 – *Continued from previous page*

Phenotype #	Phenotype name	Viable/Inviable	Changes in WT parameters
			ks_{20}=0; $ks_{20,m1}$=0; [CDC20T]=0; [CDC20A-APCP]=0; ks_{pds}=0; [PDS1T]=0;
72	*CLB5-dbΔ cdc20Δ pds1Δ*	Inviable	$kd_{b5,20}$=0; $kd_{b5,20,i}$=0; ks_{20}=0; $ks_{20,m1}$=0; [CDC20T]=0; [CDC20A-APCP]=0; ks_{pds}=0; [PDS1T]=0;
73	*CLB5-dbΔ pds1Δ*	Viable	$kd_{b5,20}$=0; $kd_{b5,20,i}$=0; ks_{pds}=0; [PDS1T]=0;
74	*GAL-CDC20*	Inviable	MDT=150; f=0.48; ks_{20}=ks_{20}*1666.67;
75	*GALL-CDC20 sic1Δ cdh1Δ*	Viable	MDT=150; f=0.48; ks_{20}=ks_{20}*1000; ks_{ki}=ks_{ki}*0.125; $ks_{ki,swi5}$=$ks_{ki,swi5}$*0.125; [CKIT]=0.2; CDH1T=0; [CDH1A]=0;
76	*GALL-CDC20 sic1Δ Cdc6Δ2-49 cdh1Δ*	Viable	MDT=150; f=0.48; ks_{20}=ks_{20}*1000; ks_{ki}=0; $ks_{ki,swi5}$=0; [CKIT]=0; [CKIP]=0; CDH1T=0; [CDH1A]=0;
77	*APC-A*	Viable	$ka_{cp,b2}$=0;
78	*APC-A sic1Δ*	Viable	$ka_{cp,b2}$=0; ks_{ki}=ks_{ki}*0.125; $ks_{ki,swi5}$=$ks_{ki,swi5}$*0.125; [CKIT]=0.2;
79	*APC-A sic1Δ cdc6Δ2-49*	Viable	$ka_{cp,b2}$=0; ks_{ki}=0; $ks_{ki,swi5}$=0; [CKIT]=0; [CKIP]=0;
80	*APC-A cdh1Δ*	Inviable	$ka_{cp,b2}$=0; CDH1T=0; [CDH1A]=0;
81	*APC-A cdh1Δ* in galactose	Inviable	$ka_{cp,b2}$=0; CDH1T=0; [CDH1A]=0; MDT=150; f=0.48;
82	*APC-A cdh1Δ GAL-SIC1*	Viable	$ka_{cp,b2}$=0; CDH1T=0; [CDH1A]=0; MDT=150; f=0.48; ks_{ki}=ks_{ki}*33.33;
83	*APC-A cdh1Δ* multicopy *SIC1*	Viable	$ka_{cp,b2}$=0; CDH1T=0; [CDH1A]=0; ks_{ki}=ks_{ki}*65; $ks_{ki,swi5}$=$ks_{ki,swi5}$*65;
84	*APC-A cdh1Δ* multicopy *CDC20*	Viable	$ka_{cp,b2}$=0; CDH1T=0; [CDH1A]=0; ks_{20}=ks_{20}*2; $ks_{20,m1}$=$ks_{20,m1}$*2;
85	*APC-A GAL-CLB2*	Inviable	$ka_{cp,b2}$=0; MDT=150; f=0.48; ks_{b2}=ks_{b2}*49.40;
86	*pds1Δ*	Viable	ks_{pds}=0; [PDS1T]=0;
87	*PDS1-dbΔ*	Inviable	$kd_{pds,20}$=0;
88	*GAL-PDS1-dbΔ*	Inviable	MDT=150; f=0.48; ks_{pds}=ks_{pds}*3.33; $kd_{pds,20}$=0;
89	*esp1-ts*	Inviable	ESP1T=0;
90	*GAL-PDS1-dbΔ esp1-ts*	Inviable	MDT=150; f=0.48; ks_{pds}=ks_{pds}*3.33; $kd_{pds,20}$=0; ESP1T=0;
91	*GAL-ESP1 cdc20-ts*	Inviable	MDT=150; f=0.48; ESP1T=ESP1T*2; ks_{20}=0; $ks_{20,m1}$=0; [CDC20T]=0; [CDC20A-APCP]=0;
92	*ppxΔ*	Viable	PPXT=0; [PPX]=0;
93	*GAL-PPX*	Viable	MDT=150; f=0.48; PPXT=PPXT*2;
94	*tem1Δ*	Inviable	TEM1T=0; [TEM1]=0;
95	*net1-ts*	Viable	kas_{net}=kas_{net}*0.45;

Continued on next page

Table S5 – *Continued from previous page*

Phenotype #	Phenotype name	Viable/Inviable	Changes in WT parameters
96	*tem1Δ net1-ts*	Viable	TEM1T=0; [TEM1]=0; kas_{net}=kas_{net}*0.45;
97	*GAL-TEM1*	Viable	TEM1T=TEM1T*5;
98	*tem1-ts GAL-CDC15*	Viable	TEM1T=0; [TEM1]=0; MDT=150; f=0.48; CDC15T=CDC15T*10;
99	*tem1-ts* multicopy *CDC14*	Viable	TEM1T=0; [TEM1]=0; CDC14T=CDC14T*2;
100	Multicopy *CDC15*	Viable	CDC15T=CDC15T*20;
101	*tem1-ts* multicopy *CDC15*	Viable	TEM1T=0; [TEM1]=0; CDC15T=CDC15T*20;
102	*net1-ts cdc20-ts*	Inviable	kas_{net}=kas_{net}*0.45; ks_{20}=0; $ks_{20,m1}$=0; [CDC20T]=0; [CDC20A-APCP]=0;
103	*cdc15Δ*	Inviable	CDC15T=0;
104	*cdc15Δ net1-ts*	Viable	CDC15T=0; kas_{net}=kas_{net}*0.45;
105	*cdc15Δ net1-ts cdh1Δ*	Viable	CDC15T=0; kas_{net}=kas_{net}*0.45; CDH1T=0; [CDH1A]=0;
106	*cdc15-ts* multicopy *TEM1*	Inviable	CDC15T=0; TEM1T=TEM1T*5;
107	*cdc15-ts* multicopy *CDC14*	Viable	CDC15T=0; CDC14T=CDC14T*2;
108	*TAB6-1*	Viable	kas_{net}=kas_{net}*0.5;
109	*cdc15Δ TAB6-1*	Viable	CDC15T=0; kas_{net}=kas_{net}*0.5;
110	*TAB6-1 clb5Δ clb6Δ*	Inviable	kas_{net}=kas_{net}*0.5; ks_{b5}=0; $ks_{b5,bf}$=0; [CLB5T]=0;
111	*TAB6-1 CLB1 clb2Δ*	Viable	kas_{net}=kas_{net}*0.5; ks_{b2}=ks_{b2}*0.33; $ks_{b2,m1}$=$ks_{b2,m1}$*0.33;
112	*cdc14-ts*	Inviable	CDC14T=0;
113	*cdc14-ts sic1Δ*	Inviable	CDC14T=0; ks_{ki}=ks_{ki}*0.125; $ks_{ki,swi5}$=$ks_{ki,swi5}$*0.125; [CKIT]=0.2;
114	*cdc14-ts cdh1Δ*	Inviable	CDC14T=0; CDH1T=0; [CDH1A]=0;
115	*cdc14-ts GAL-SIC1*	Inviable	CDC14T=0; MDT=150; f=0.48; ks_{ki}=ks_{ki}*33.33;
116	*cdc14-ts GAL-CLN2*	Inviable	CDC14T=0; MDT=150; f=0.48; ks_{n2}=0.15;
117	*GAL-NET1*	Inviable	MDT=150; f=0.48; NET1T=NET1T*10.85;
118	*GAL-CDC14*	Inviable	MDT=150; f=0.48; CDC14T=CDC14T*7;
119	*GAL-NET1 GAL-CDC14*	Viable	MDT=150; f=0.48; CDC14T=CDC14T*7; NET1T=NET1T*10.85;

Table S6: **Optimization results with different hypercube sizes**

Hypercube size	Mean number of hits	Standard deviation
±40%	105.42	2.48
±20%	100.10	2.40
±90%	103.98	2.87

Mean and standard deviation values of the number of hits reached after 500 generations with 3 different hypercube sizes (50 independent runs for each size). All LH samples are generated from the TL parameter set with 72 hits. Population size is 19, initial populations are independently generated for each run without enforcing wild type viability in glucose.

Table S7: **Eight phenotypes that are not captured by the best DE run**

Phenotype number	Phenotype name	$\overline{A}$ (100 LH samples)	$\overline{A}$ (19,000 LH samples)	$\overline{A}$ (19,000 DE samples)	$\hat{R}$ rank (100 LH samples)	$\hat{R}$ rank (19,000 LH samples)	$\hat{R}$ rank (19,000 DE samples)
12	*cln1*Δ *cln2*Δ *bck2*Δ (viable)	0.03	0.05	0.06	48	58	6
46	*CLB5-db*Δ *sic1*Δ (inviable)	0.94	0.92	0.09	3	12	1
48	*GAL-CLB5 sic1*Δ (inviable)	0.88	0.79	0.08	22	9	3
55	*GAL-CLB2 sic1*Δ (inviable)	0.86	0.83	0.08	13	11	2
66	*CLB1 clb2*Δ *cdh1*Δ (inviable)	0.57	0.52	0.08	5	6	5
67	*CLB1 clb2*Δ *pds1*Δ (inviable)	0.36	0.35	0.01	2	2	7
74	*GAL-CDC20* (inviable)	0.41	0.37	0.01	4	4	8
117	*GAL-NET1* (inviable)	0.52	0.52	0.01	1	1	4

Features of the eight phenotypes not captured by DE in the most optimal run that resulted in 111 hits. $\overline{A}$ stands for the acceptance ratio of a phenotype, $\hat{R}$ rank is based on the phenotype competitiveness (most competitive phenotype, with the lowest $\hat{R}$ value, is ranked 1st).

Table S8: **Most competitive phenotype pairs in LH samples**

Phenotype pair	Correlation coefficient	Acceptance ratio
cln1Δ cln2Δ (viable)<br>*cln1Δ cln2Δ cdh1Δ GAL-SIC1* (inviable)	-0.846	0.07<br>0.93
CLB2-dbΔ in galactose (inviable)<br>*CLB2-dbΔ clb5Δ clb6Δ* in galactose (viable)	-0.808	0.94<br>0.04
WT in galactose (viable)<br>*GAL-NET1* (inviable)	-0.698	0.31<br>0.52
CLB5-dbΔ sic1Δ (inviable)<br>*cln1Δ cln2Δ bck2Δ* (viable)	-0.696	0.94<br>0.03
APC-A cdh1Δ in galactose (inviable)<br>*APC-A cdh1Δ GAL-SIC1* (viable)	-0.657	0.88<br>0.11
cln1Δ cln2Δ bck2Δ (viable)<br>*cln1Δ cln2Δ GAL-SIC1* (inviable)	-0.656	0.03<br>0.97
cln1Δ cln2Δ bck2Δ (viable)<br>*CLB2-dbΔ* (inviable)	-0.656	0.03<br>0.97
sic1Δ (viable)<br>*CLB5-dbΔ sic1Δ* (inviable)	-0.645	0.06<br>0.94
cln1Δ cln2Δ bck2Δ (viable)<br>*cln1Δ cln2Δ cdh1Δ GAL-SIC1* (inviable)	-0.641	0.03<br>0.93
sic1Δ (viable)<br>*GAL-CLB2 sic1Δ* (inviable)	-0.626	0.06<br>0.86
CLB2-dbΔ in galactose (inviable)<br>*APC-A* (viable)	-0.618	0.94<br>0.10
cdh1Δ (viable)<br>*GAL-CLB5 cdh1Δ* (inviable)	-0.607	0.12<br>0.73
GAL-PPX (viable)<br>*GAL-NET1* (inviable)	-0.601	0.25<br>0.52

Strongly anticorrelated phenotype pairs (correlation coefficient < -0.6) computed from 100 LH samples.

Table S9: **Most competitive phenotype pairs in DE samples**

Phenotype pair	Correlation coefficient	Acceptance ratio
GAL-NET1 (inviable)<br>*GAL-PPX* (viable)	-0.812	0.01<br>0.98
GAL-NET1 (inviable)<br>WT in galactose (viable)	-0.806	0.01<br>0.99
GAL-NET1 (inviable)<br>*GAL-TEM1* (viable)	-0.765	0.01<br>0.98
GAL-NET1 (inviable)<br>WT in glucose (viable)	-0.763	0.01<br>0.98
GAL-NET1 (inviable)<br>Multicopy *CDC15* (viable)	-0.739	0.01<br>0.98
*CLB5-db*Δ *sic1*Δ (inviable)<br>*cln1*Δ *cln2*Δ *sic1*Δ (viable)	-0.729	0.09<br>0.89
GAL-NET1 (inviable)<br>Multicopy *BCK2* (viable)	-0.694	0.01<br>0.98
*CLB5-db*Δ *sic1*Δ (inviable)<br>*swi5*Δ (viable)	-0.675	0.09<br>0.91
*CLB5-db*Δ *sic1*Δ (inviable)<br>*sic1*Δ (viable)	-0.671	0.09<br>0.91
GAL-NET1 (inviable)<br>*GAL-CLN3* (viable)	-0.661	0.01<br>0.99
*CLB5-db*Δ *sic1*Δ (inviable)<br>*APC-A sic1*Δ *cdc6*Δ*2-49* (viable)	-0.661	0.09<br>0.87
*CLB5-db*Δ *sic1*Δ (inviable)<br>*cln1*Δ *cln2*Δ *cki*Δ (viable)	-0.654	0.09<br>0.91
*CLB5-db*Δ *sic1*Δ (inviable)<br>*sic1*Δ *cdc6*Δ (*cki*Δ) (viable)	-0.646	0.09<br>0.90
*cln1*Δ *cln2*Δ *sic1*Δ (viable)<br>*GAL-CLB2 sic1*Δ (inviable)	-0.646	0.89<br>0.08
*swi5*Δ (viable)<br>*GAL-CLB2 sic1*Δ (inviable)	-0.634	0.91<br>0.08
GAL-NET1 (inviable)<br>*cln1*Δ *cln2*Δ *GAL-CLN2* (viable)	-0.629	0.01<br>0.98
*sic1*Δ (viable)<br>*GAL-CLB2 sic1*Δ (inviable)	-0.627	0.91<br>0.08
GAL-NET1 (inviable)<br>*cln3*Δ *bck2*Δ *whi5*Δ (viable)	-0.615	0.01<br>0.98
GAL-NET1 (inviable)<br>*tem1-ts* multicopy *CDC15* (viable)	-0.612	0.01<br>0.97

Strongly anticorrelated phenotype pairs (correlation coefficient < -0.6) among 19,000 unique trial parameter vectors generated during DE.

Table S10: **Least and most competitive phenotypes in LH samples**

**Least competitive phenotypes**	$\hat{R}$		**Most competitive phenotypes**	$\hat{R}$
Multicopy *CDC15* (viable)	13.71		*GAL-NET1* (**Phenotype 117**, inviable)	-8.32
WT in galactose (viable)	13.31		*CLB1 clb2Δ pds1Δ* (**Phenotype 67**, inviable)	-7.86
CLB5-dbΔ (viable)	12.93		*CLB5-dbΔ sic1Δ* (**Phenotype 46**, inviable)	-6.16
GAL-TEM1 (viable)	12.78		*GAL-CDC20* (**Phenotype 74**, inviable)	-5.88
WT in glucose (viable)	12.78		*CLB1 clb2Δ cdh1Δ* (**Phenotype 66**, inviable)	-4.81
cln3Δ bck2Δ whi5Δ (viable)	11.83		*GAL-CLB5 cdh1Δ* (inviable)	-4.29
GAL-SIC1 (viable)	11.41		*tem1Δ* (inviable)	-3.75
GAL-CLN3 (viable)	11.35		*cdc15Δ* (inviable)	-3.75
Multicopy *BCK2* (viable)	11.11		*cdc15-ts* multicopy *TEM1* (inviable)	-3.75
swi5Δ cdh1Δ GAL-SIC1 (viable)	10.76		*cln1Δ cln2Δ GAL-SIC1* (inviable)	-3.39
GAL-PPX (viable)	9.83		*cln1Δ cln2Δ sic1Δ* (viable)	-3.24
cln1Δ cln2Δ GAL-CLN2 (viable)	9.28		*APC-A sic1Δ* (viable)	-3.24
cln1Δ cln2Δ cln3Δ multicopy *BCK2* (viable)	9.21		*GAL-CLB2 sic1Δ* (**Phenotype 55**, inviable)	-3.00
GAL-CLB2 (viable)	8.96		*cln1Δ cln2Δ cdh1Δ GAL-SIC1* (inviable)	-2.94
CLB5-dbΔ pds1Δ (viable)	8.88		*TAB6-1 clb5Δ clb6Δ* (inviable)	-2.92
TAB6-1 (viable)	8.85		*cdc20Δ pds1Δ* (inviable)	-2.87
cln1Δ cln2Δ cln3Δ GAL-CLN2 (viable)	8.73		*CLB5-dbΔ cdc20Δ pds1Δ* (inviable)	-2.87
cln1Δ cln2Δ cln3Δ bck2Δ GAL-CLN2 (viable)	8.68		*GAL-CLB5-dbΔ* (inviable)	-2.75
cln1Δ cln2Δ cln3Δ GAL-CLN3 (viable)	8.59		*GAL-ESP1 cdc20-ts* (inviable)	-2.22
CLB1 clb2Δ (viable)	8.58		*CLB2-dbΔ clb5Δ clb6Δ* in galactose (viable)	-2.04
cln1Δ cln2Δ GAL-SIC1 GAL-CLN2 (viable)	8.55		*GAL-CLB2 cdh1Δ* (inviable)	-1.98
pds1Δ (viable)	8.37		*GAL-CLB5 sic1Δ* (**Phenotype 48**, inviable)	-1.96
cln1Δ cln2Δ cdh1Δ GAL-SIC1 GAL-CLN2 (viable)	8.16			
net1-ts (viable)	7.81			
GAL-CLB5 (viable)	7.32			
clb5Δ clb6Δ (viable)	7.15			
GAL-NET1 GAL-CDC14 (viable)	7.08			
APC-A cdh1Δ multicopy *SIC1* (viable)	7.01			
net1-ts cdc20-ts (inviable)	6.84			
GAL-CLB2-dbΔ (inviable)	6.84			

The least competitive 30 phenotypes and the most competitive 22 phenotypes based on the $\hat{R}$ values (defined in the main text) computed from 100 LH samples. The most competitive phenotype has the lowest $\hat{R}$ value, whereas the least competitive phenotype has the highest $\hat{R}$ value. When 19,000 independently generated LH samples were taken, 26 of the least competitive 30 phenotypes were common. Similarly, all of the 22 most competitive phenotypes were common among 100 and 19,000 LH samples. Phenotype numbers for the 8 phenotypes that are not captured by the best DE run are marked in bold.

Table S11: **The least critical model parameters**

Parameter name	Parameter description
$ks_{bud,e}$	Time scale for BUD synthesis
$e_{bud,n3}$	Cln3 activation of BUD
$e_{bud,n2}$	Cln2 activation of BUD
$e_{bud,b5}$	Clb5 activation of BUD
$e_{bud,b2}$	Clb2 activation of BUD
kd_{bud}	BUD degradation
$[\text{Cln3}]_0$	Initial Cln3
$[\text{Bck2}]_0$	Initial Bck2
$[\text{WHI5dep}]_0$	Initial active Whi5
$[\text{SBFdep}]_0$	Initial active SBF
$[\text{Cln2}]_0$	Initial Cln2
$[\text{CKI}_T]_0$	Initial active CKI
$[[\text{CKI}_P]_0$	Initial inactive CKI
$[\text{Clb5}_T]_0$	Initial Clb5
$[\text{Clb2}_T]_0$	Initial Clb2
$[\text{BUD}]_0$	Initial BUD
$[\text{ORI}]_0$	Initial ORI
$[\text{SPN}]_0$	Initial SPN
$[\text{Swi5}_T]_0$	Initial SWI5
$[CDC20_T]_0$	Initial Cdc20
$[\text{Cdc20A-APCP}]_0$	Initial Cdc20A-APCP
$[\text{APCP}]_0$	Initial APCP
$[\text{Cdh1}_A]_0$	Initial active Cdh1
$[\text{Net1dep}]_0$	Initial active Net1
$[\text{PPX}]_0$	Initial PPX
$[\text{Pds1}_T]_0$	Initial Pds1
$[\text{Cdc15}]_0$	Initial Cdc15
$[\text{Tem1}]_0$	Initial Tem1
$[\text{Polo}_T]_0$	Initial Polo
$[\text{Polo}_A]_0$	Initial active Polo
$[\text{Cdc20A-APC}]_0$	Initial Cdc20A-APC
$e_{h1,n3}$	Cdh1 inactivation by Cln3
ka_{tem}	Basal Tem1 activation
ka_{15}	Basal Cdc15 activation
ks_{lo}	Basal Polo synthesis
kd_{ori}	Degradation of ORI
$ka_{tem,p1}$	Tem1 activation by Esp1
ka_{lo}	Basal Polo activation
$ki_{tem,px}$	Tem1 inactivation by PPX
kp_{i5b5}	Whi5 phosphorylation by Clb5
$ki_{15,b2}$	Cdc15 inactivation by Clb2
ki_{h1}	Basal inactivation of Cdh1
$kd_{lo,h1}$	Polo degradation by Cdh1
ki_{px}	Basal PPX inactivation
$kdp_{net,14}$	Net1 dephosphorylation by Cdc14
kd_{lo}	Basal Polo degradation
$kp_{net,15}$	Net1 phosphorylation by Cdc15
$ki_{swi5,b2}$	Swi5 inactivation by Clb2
$e_{ki,k2}$	CKI phosphorylation by Bck2
kdp_{i514}	Whi5 dephosphorylation by Cdc14

50 least critical model parameters that do not have an influence on the model's ability to capture mutant phenotypes.
