# Supplementary material for: Optimization and model reduction in the high dimensional parameter space of a budding yeast cell cycle model
Source: BMC Syst Biol. 2013 Jun 28;7:53. doi: 10.1186/1752-0509-7-53 (PMC3702416; doi:10.1186/1752-0509-7-53)
Supplement: Additional file 3 — Supplementary Text. This pdf file includes a detailed description of Latin Hypercube sampling. [file 1752-0509-7-53-S3.pdf]

Supplementary Text

Latin hypercube sampling

Latin hypercube (LH) sampling is a method [1] for generating a distribution of parameter sets from a multidimensional distribution:

- Let x represent a D dimensional parameter vector $x = x_1, x_2, ..., x_D$. D is also the number of parameters in our ODE model.

- When sampling for M parameter vectors (sets), the range of each parameter is divided into $M > 1$ mutually exclusive intervals.

- One sample point is randomly selected from each interval for the first parameter x_1. Hence, M values are sampled: $x_{1,1}$ through $x_{1,M}$. These values are paired with M values sampled for the second parameter x_2 randomly without replacement, which means that each x_2 value is paired with only one value of x_1.

- Then, these M pairs are combined with M values of x_3, once again without replacement and M triplets are obtained.

- This procedure is continued until M D-tuplets are generated.

LHS provides a fairly uniform coverage of each parameter range by dividing each range into a number of intervals. The number of intervals is equal to the number of samples.

References

1. Santner TJ, Williams B, Notz W: *The Design and Analysis of Computer Experiments*. Springer-Verlag 2003.
