# Supplementary material for: Optimization and model reduction in the high dimensional parameter space of a budding yeast cell cycle model
Source: BMC Syst Biol. 2013 Jun 28;7:53. doi: 10.1186/1752-0509-7-53 (PMC3702416; doi:10.1186/1752-0509-7-53)
Supplement: Additional file 4 — Supplementary Figures. This pdf file includes two additional figures. [file 1752-0509-7-53-S4.pdf]

Supplementary Figures

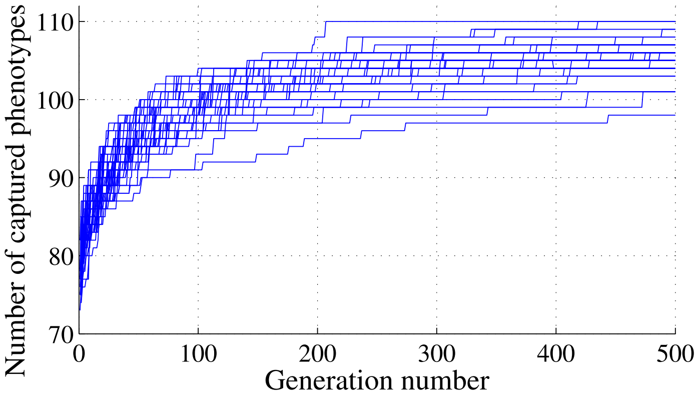

Figure S1. Evolution of the number of mutants captured with 50 independent DE runs. Each run starts with a different initial population of 19 LH samples (independently generated populations with ±40% LH sampling, wild type viability not enforced). The number of phenotypes captured after 500 generations has a 105.42 mean and 2.48 standard deviation (Table S6).

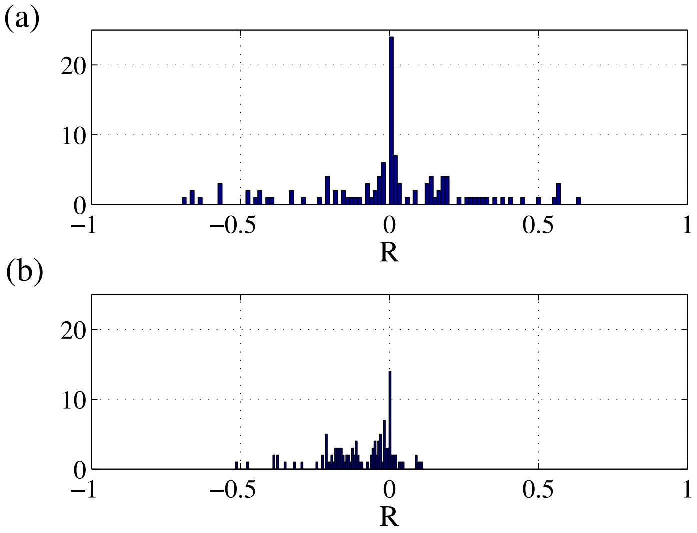

Figure S2. Distribution of the pairwise correlations (R values) of Phenotype 12 with the remaining 118 phenotypes in (a) 100 LH samples and (b) 19,000 DE samples (trial parameter vectors).
